# Polymer‐Assisted Direct and Rapid Microwave Synthesis of Mesoporous Binary and Ternary Metal Oxides for Electrocatalytic Water Oxidation

**DOI:** 10.1002/smll.202510771

**Published:** 2025-12-17

**Authors:** Jasmin Helgert, Jana Timm, Lion Schumacher, Roland Marschall

**Affiliations:** ^1^ Physical Chemistry III University of Bayreuth 95447 Bayreuth Germany

**Keywords:** electrochemistry, mesoporous materials, metal oxides, microwave synthesis, water splitting

## Abstract

A novel quick and facile polymer‐assisted microwave synthesis route to prepare mesoporous binary metal oxides α‐Fe_2_O_3_ and α‐Mn_2_O_3_ and spinel‐type ferrites NiFe_2_O_4_ and ZnFe_2_O_4_ is presented, which can potentially be applied for many other mixed metal oxides. The presented synthesis only needs 15–30 min, much shorter than conventional approaches for mesoporous materials. Thorough characterization of the materials is performed by Powder X‐Ray Diffraction (PXRD), Raman spectroscopy, energy dispersive X‐ray spectroscopy (EDXS), nitrogen physisorption analysis, mercury intrusion porosimetry (MIP), diffuse reflectance infrared fourier transform (DRIFT) spectroscopy, UV–Vis‐spectroscopy, X‐Ray photoelectron spectroscopy (XPS), and scanning (SEM) as well as transmission electron microscopy (TEM) and selected area electron diffraction (SAED). Furthermore, mesoporous α‐Mn_2_O_3_ and NiFe_2_O_4_ are applied as electrocatalysts for electrocatalytic oxygen evolution in alkaline media, showing improved performance compared to nanoparticles or EISA‐derived mesoporous NiFe_2_O_4_.

## Graphical abstract

A novel direct and facile microwave synthesis route to prepare mesoporous binary and ternary metal oxides with P‐123^®^ as porogen is presented. NiFe_2_O_4_ is applied in alkaline Oxygen Evolution Reaction (OER) and shows improved performance in comparison to nanoparticular and  Evaporation Induced Self‐Assembly (EISA)‐derived mesoporous NiFe_2_O_4_.

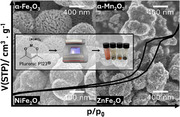

## 1 Introduction

As the global demand for energy rises, the search for sustainable energy sources as well as the storage of energy is crucial.^[^ ^1^ ^]^ Hydrogen is widely regarded as an ideal alternative energy source due to its high energy density, low molecular weight, and clean and pollution‐free properties. Electrocatalytic water splitting is considered to be a promising approach for converting electrical energy into hydrogen, offering a sustainable and environmentally friendly way to store renewable energy.^[^ ^2^, ^3^, ^4^ ^]^ Despite its potential, water splitting is hindered by the reaction kinetics of the oxygen evolution reaction (OER). The introduction of efficient electrocatalysts can substantially lower the overpotential required for OER, thereby enhancing the overall reaction efficiency.^[^ ^5^ ^]^

Since the metals of state‐of‐the‐art electrocatalysts for OER such as RuO_2_ and IrO_2_ are limited in their availability and come with high manufacturing costs, 3D transition metal oxides with earth‐abundant metals such as iron, zinc, nickel or manganese have been extensively studied as OER electrocatalysts.^[^ ^4^, ^6^ ^]^ Manganese oxides are considered promising electrocatalysts for OER due to the existence of numerous structures and morphologies, with an electronic structure favourable for OER.^[^ ^4^, ^7^ ^]^ Gorlin and Jaramillo^[^ ^8^ ^]^ reported on a manganese oxide film capable of OER as well as oxygen reduction reaction. However, manganese‐based compounds often suffer from poor stability^[^ ^9^ ^]^ and low activity.^[^ ^4^ ^]^

With iron being the fourth most abundant element on earth, iron based materials such as binary iron oxides maghemite, magnetite and hematite or ternary metal oxides, namely spinel ferrites, are of interest and can be applied in a wide range of fields, such as catalysis, biomedicine, environmental remediation and energy storage devices, owing to their intrinsic and unique properties.^[^ ^10^, ^11^ ^]^ Spinel‐type ferrites with the general stoichiometric formula MFe_2_O_4_ with M being a divalent metal ion such as Mn^2+^, Ni^2+^, Cu^2+^, Co^2+^, Zn^2+^ are well investigated compounds, with NiFe_2_O_4_ being considered to be an efficient OER electrocatalyst in alkaline media.^[^ ^12^, ^13^, ^14^, ^15^ ^]^ Oxide ions in the normal spinel form a cubic close‐packed lattice and the divalent cations occupy 1/8 of the tetrahedral vacancies whereas the trivalent cations occupy 2/4 of the octahedral vacancies. In contrast to the normal spinel the divalent cations in the inverse spinel occupy 1/4 of the octahedral vacancies and the trivalent cations occupy 1/8 of the tetrahedral vacancies and 1/4 of the octahedral vacancies.^[^ ^16^ ^]^ The inversion parameter λ describes the cationic distributions in tetrahedral and octahedral sites. Whereas for NiFe_2_O_4_ an inversion degree of λ = 1 (inverse spinel) is expected,^[^ ^14^, ^17^, ^18^ ^]^ ZnFe_2_O_4_ exhibits a normal spinel (λ = 0) structure in bulk form at room temperature, while an inverted spinel structure has been observed at the nanoscale.^[^ ^19^ ^]^ ZnFe_2_O_4_ is a semiconductor with a narrow band gap of 1.9 eV which can be applied in catalysis^[^ ^20^ ^]^ and is also considered to be a promising anode material for lithium ion batteries (LIBs).^[^ ^21^ ^]^ Hematite, α‐Fe_2_O_3_, can also be applied in the area of catalysis as an n‐type semiconductor for photocatalytic OER^[^ ^22^ ^]^ and is also assumed to be a promising anode material for LIBs.^[^ ^23^ ^]^

Materials with high surface area, large pore volume and tailorable surface properties are attracting widespread attention for adsorption and catalysis applications. By nanostructuring the performance of an electrocatalyst can be enhanced due to a more accessible active surface area whereas the sizes of the pores influence mass transport characteristics.^[^ ^24^, ^25^ ^]^ In addition, the diffusion paths of charge carriers in semiconductors can be shortened by nanostructuring as many transition metal oxides are poorly conductive.^[^ ^26^ ^]^

According to IUPAC mesoporous materials include substances with pore sizes of 2 nm up to 50 nm. Substances with smaller pore sizes are classified as microporous, and materials with pore sizes bigger than 50 nm are classified as macroporous.^[^ ^27^ ^]^ The preparation of ordered porous materials has attracted considerable interest since the first report of the synthesis of ordered mesoporous silica M41S by Mobil researchers in 1992.^[^ ^28^ ^]^ Various synthetic strategies to prepare mesoporous materials have been employed, ranging from soft and hard templating^[^ ^29^ ^]^ to combustion synthesis.^[^ ^30^ ^]^ Combustion synthesis is based on the exothermic redox reaction between metal nitrates and a suitable fuel. The formation of the porous morphology is attributed to the sudden release of gas during the combustion process.^[^ ^30^ ^]^ Another solution‐based synthesis method is the complexation process in which chelating agents such as citric acid or ethylenediaminetetraacetic acid are used to chelate metal cations in solution.^[^ ^31^ ^]^ Thermal decomposition of a suitable precursor (organometallic compounds or metal‐surfactant complexes) can also lead to porous morphologies.^[^ ^32^ ^]^

Regarding the aforementioned catalyst materials, literature reports on mesoporosity are quite numerous. For example, Simon et al.^[^ ^13^ ^]^ presented a soft‐templating route for the synthesis of ordered mesoporous NiFe_2_O_4_, based on the Evaporation Induced Self‐Assembly (EISA) route with citric acid and in the absence and presence of Pluronic P‐123 with tuneable pore sizes in the range of 5–12 nm depending on the calcination process. Yen et al.^[^ ^33^ ^]^ reported on the synthesis of ordered mesoporous NiFe_2_O_4_ by using nitrate salts as precursors and different mesoporous silicas (MCM‐41, SBA‐15, KIT‐6) as hard templates. Jiao et al.^[^ ^34^ ^]^ performed hard‐templating using silica KIT‐6 and obtained unordered and ordered crystalline α‐Fe_2_O_3_ depending on synthesis conditions with pore diameters ≈3.8 nm. Jiao and Bruce^[^ ^35^ ^]^ also synthesized ordered mesoporous hematite via a soft‐templating route with 2D and 3D pores using decylamine as a template and iron(III)ethoxide as precursor. Zhou et al.^[^ ^36^ ^]^ employed a hydrothermal route with ethanol‐ethylene glycol as a binary solvent for the preparation of ZnFe_2_O_4_. After annealing at 400 °C for 2 h, porous ZnFe_2_O_4_ nanospheres with an average diameter ≈230 nm were obtained. Li et al.^[^ ^37^ ^]^ reported on the synthesis of ZnFe_2_O_4_ nanorods with porous surface, starting from hydrothermally prepared zinc oxalate ZnFe_2_(C_2_O_4_)_3_ as self‐template. Mesoporous α‐Mn_2_O_3_ was prepared by Sa et al.^[^ ^38^ ^]^ by hard‐templating with silica‐based KIT‐6, whereas Piumetti et al.^[^ ^39^ ^]^ employed solution combustion synthesis of porous α‐Mn_2_O_3._

In contrast to previously introduced methods for the preparation of mesoporous materials, microwave synthesis stands out especially regarding the shortened reaction time as well as the quick preparation of the precursor solution. In contrast, silica‐based hard‐templates might introduce impurities of insulating SiO_2_ whereas hydrothermal treatment or the EISA process is often performed for extensive time spans.^[^ ^13^, ^36^ ^]^ Microwave synthesis enables fast and reproducible synthesis of materials by efficient and controlled heating provided by microwave irradiation whereas combustion synthesis is often performed under harsh conditions.^[^ ^39^ ^]^ Dolcet et al.^[^ ^40^ ^]^ performed synthesis of ZnFe_2_O_4_ via a hydrothermal, a combined miniemulsion/hydrothermal and microwave synthesis route and found that ZnFe_2_O_4_ was formed after 5 min only during the microwave approach. The reaction parameters such as temperature (during heating and synthesis) and pressure, using pressurized reaction vessels, in microwave synthesis are well controlled and monitored, thus side reactions can be suppressed. Furthermore, the reaction solution is not in direct contact with the heating source.^[^ ^41^ ^]^ A well‐designed vessel provides a constant temperature increase over the sample, resulting in fewer side‐products and/or product degradation.^[^ ^42^ ^]^

To the best of our knowledge, a direct microwave synthesis of mesoporous materials with commercially available block copolymer Pluronic P‐123 has not been reported yet. Here, we report a novel, efficient, and straightforward microwave synthesis method to prepare mesoporous binary metal oxides (α‐Fe_2_O_3_ and α‐Mn_2_O_3_) and spinel‐type ferrites (NiFe_2_O_4_ and ZnFe_2_O_4_) with Pluronic P‐123 as porogen. This synthesis method requires a significantly shorter synthesis time than conventional preparation of mesoporous materials, with a synthesis time of only 15–30 min. A comprehensive characterisation process was conducted, including thorough pore analysis of the materials. Mesoporous α‐Mn_2_O_3_ and NiFe_2_O_4_ were utilized as electrocatalysts for the electrocatalytic OER in alkaline media. This study shows that the utilisation of microwave synthesis for the preparation of mesoporous electrocatalysts results in the generation of materials with superior performance in comparison to nanoparticle‐based and EISA‐derived mesoporous electrocatalysts. The simplicity of this synthesis method suggests numerous potential applications for a wide range of metal oxides.

## 2 Results and Discussion

**Scheme 1:**
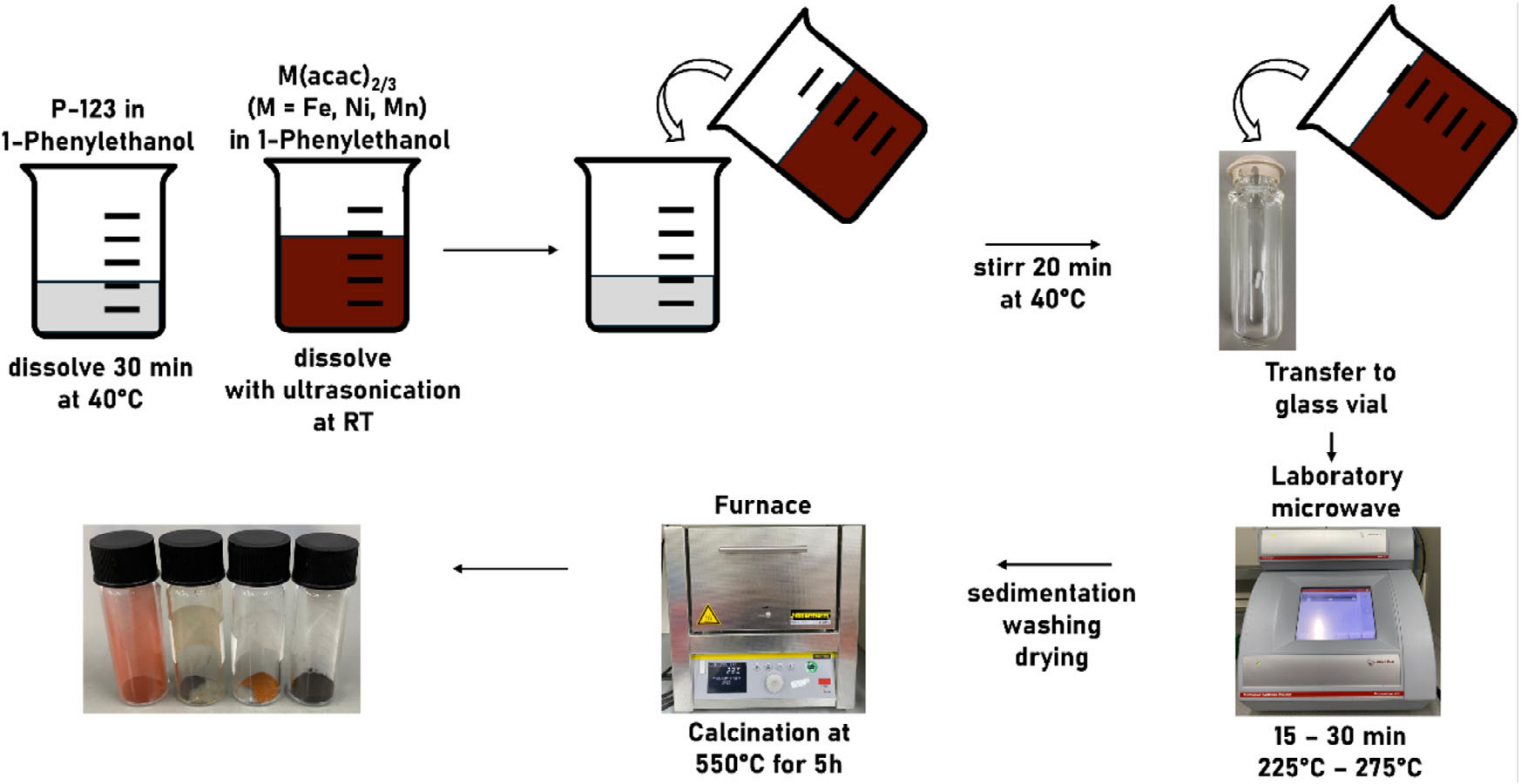
Synthesis procedure of microwave‐derived mesoporous metal oxides.

Mesoporous materials were prepared by water‐free microwave‐assisted synthesis starting from metal acetylacetonates dissolved in *rac*‐1‐phenylethanol, adapted according to the mechanism proposed by Niederberger and Garnweitner.^[^ ^43^ ^]^ A schematic overview of the synthesis procedure is shown in **Scheme** **1**. The porogen Pluronic P‐123 was dissolved at 40 °C in *rac*‐1‐phenylethanol whereas the precursor solution was prepared at room temperature (RT). After unification of the solutions, the mixture was treated in the microwave at a set temperature for a set time span. The ideal reaction temperature and reaction time was determined by variation of these parameters. Parameters for the synthesis of nanoparticles of spinel‐type NiFe_2_O_4_ and ZnFe_2_O_4_ were adopted from the literature.^[^ ^14^, ^40^ ^]^ Subsequent calcination at 550 °C was executed to remove organic precursor residues as well as Pluronic P‐123.

**Figure 1:**
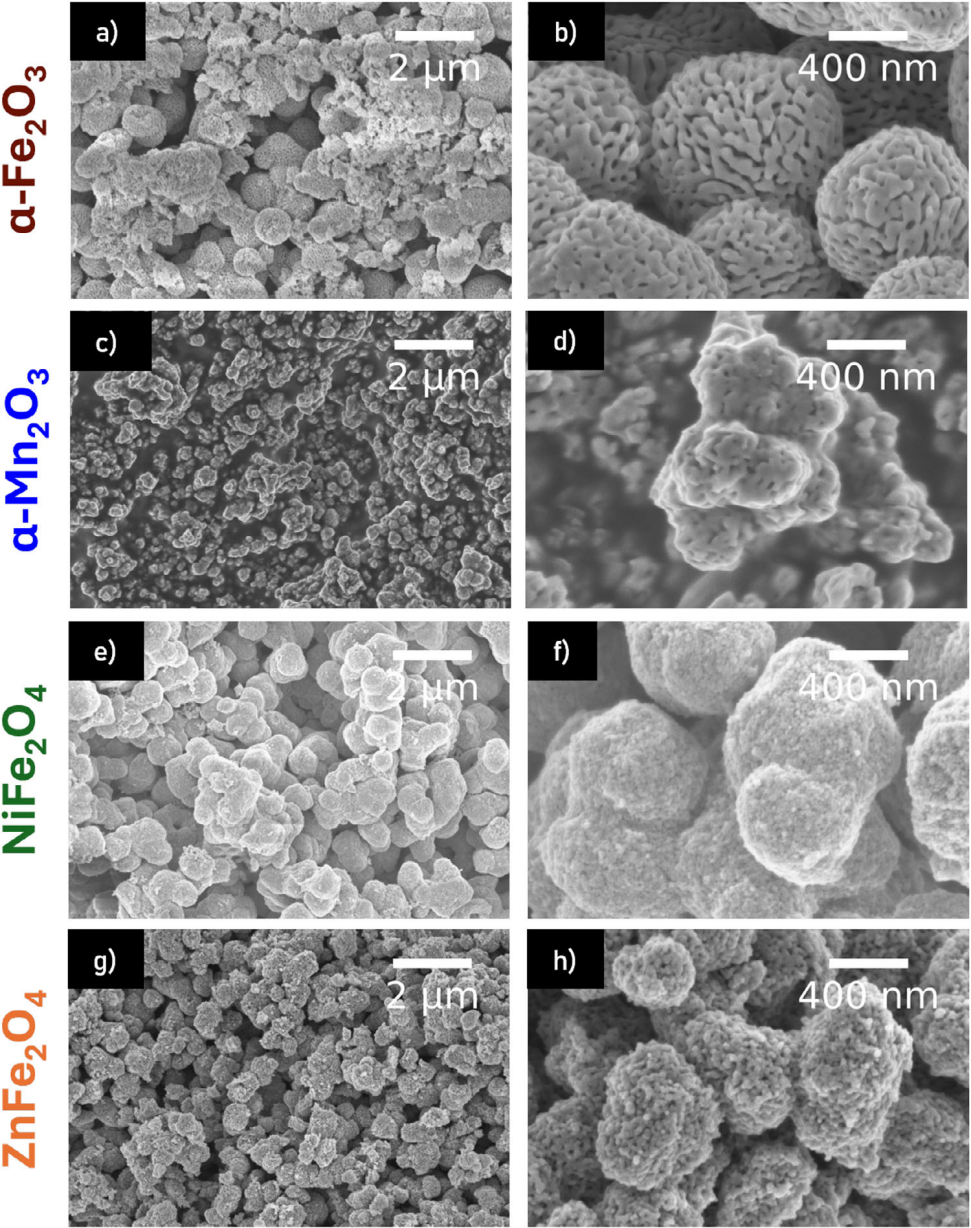
Overview (left) and close up (right) SEM images of porous a), b) α‐Fe_2_O_3,_ c), d) α‐Mn_2_O_3,_ e), f) NiFe_2_O_4_ and g), h) ZnFe_2_O_4._

SEM images of the mesoporous samples (**Figure** **1**) reveal that the calcined samples exhibit a spherical morphology with particles in the range of several hundred nanometres (600–800 nm), with especially α‐Fe_2_O_3_ displaying highly uniform spheres. Particles of α‐Mn_2_O_3_ seem rather irregular instead. In case of α‐Fe_2_O_3_ and α‐Mn_2_O_3_ completely intergrown structures could be observed, while the structure of NiFe_2_O_4_ and ZnFe_2_O_4_ resemble connected particles. Nevertheless, all compounds display a morphology which might be best described as sponge‐like with unordered pores.

**Figure 2:**
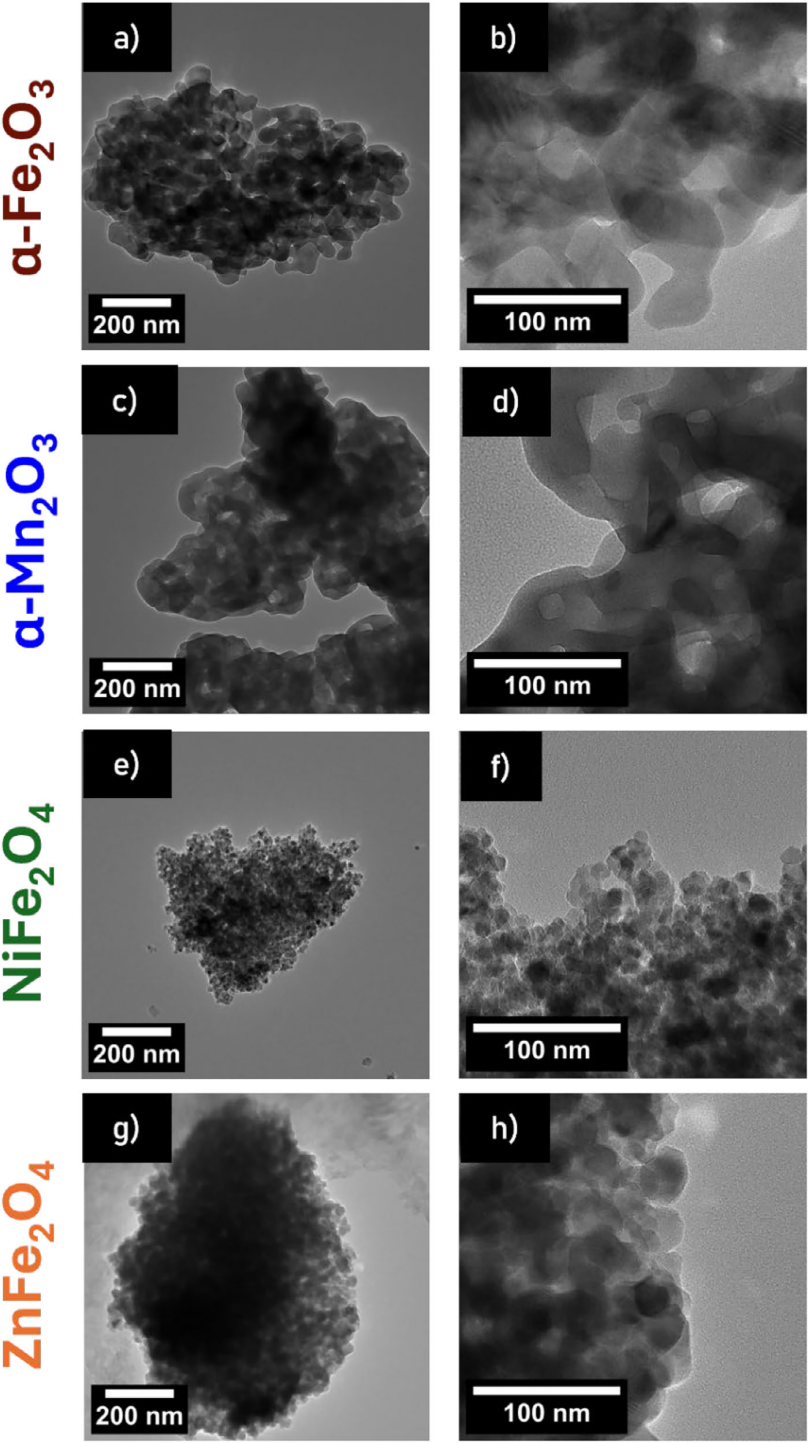
TEM images of porous a,b) α‐Fe_2_O_3,_ c,d) α‐Mn_2_O_3,_ e,f) NiFe_2_O_4_ and g,h) ZnFe_2_O_4_.

TEM images of the respective compounds are presented in **Figure** **2**, and the corresponding SAED patterns and d‐spacings are given in Figure S1 (Supporting Information), which are in accordance with literature.^[^ ^14^, ^44^, ^45^ ^]^

Townsend et al.^[^ ^22^ ^]^ prepared nanoparticles as well as bulk material of α‐Fe_2_O_3_. The morphology of particles presented in the respective TEM images (Figure 2 vs Townsend et al.^[^ ^22^ ^]^) is clearly different from mesoporous α‐Fe_2_O_3_ prepared in this work. Hierarchical spherical structures with changing material contrast, indicating high porosity, are visible in Figure 2a as well as isolated particles with holes due to the removal of P‐123. Intertwined porous structures can be observed for α‐Mn_2_O_3_, which contrast singular α‐Mn_2_O_3_ nanoparticles, as prepared by, e.g., Fardood et al.^[^ ^46^ ^]^ Synthesis of α‐Mn_2_O_3_ and α‐Fe_2_O_3_ without P‐123 was conducted as a reference and the results are summarized in Figure S2 (Supporting Information). Whereas for α‐Fe_2_O_3_ without P‐123 a similar morphology is obtained, α‐Mn_2_O_3_ without P‐123 exhibits smaller non‐porous particles.

For NiFe_2_O_4_ and ZnFe_2_O_4_ large particulate structures with changing material contrast are visible in Figure 2. A temperature‐dependent study on the morphology of ferrites NiFe_2_O_4_ and ZnFe_2_O_4_ is presented in Figures S4 and S6 (Supporting Information). SEM images demonstrate that the spherical mesoporous structure is only formed starting from a temperature of 255 °C for ZnFe_2_O_4_ and in a temperature range of 240–245 °C for NiFe_2_O_4_. Nanoparticles of NiFe_2_O_4_ and ZnFe_2_O_4_ were also prepared according to the procedures described by Simon et al.^[^ ^14^ ^]^ and Dolcet et al.^[^ ^40^ ^]^ with the respective data available as comparison (Figures S4 and S6, Supporting Information). SEM images of mesoporous NiFe_2_O_4_ and ZnFe_2_O_4_ clearly differ from SEM images of nanoparticular NiFe_2_O_4_ and ZnFe_2_O_4_. As shown in Figures S4 and S6 (Supporting Information), as well as in the Supporting Information of Simon et al,^[^ ^14^ ^]^ nanoparticles tend to form bulky agglomerates in contrast to the spherical morphology obtained for NiFe_2_O_4_ presented in this work. ZnFe_2_O_4_ nanoparticles prepared at 275 °C also agglomerate in spherical shape but the sponge‐like morphology is only visible for ZnFe_2_O_4_ prepared with P‐123.

**Figure 3:**
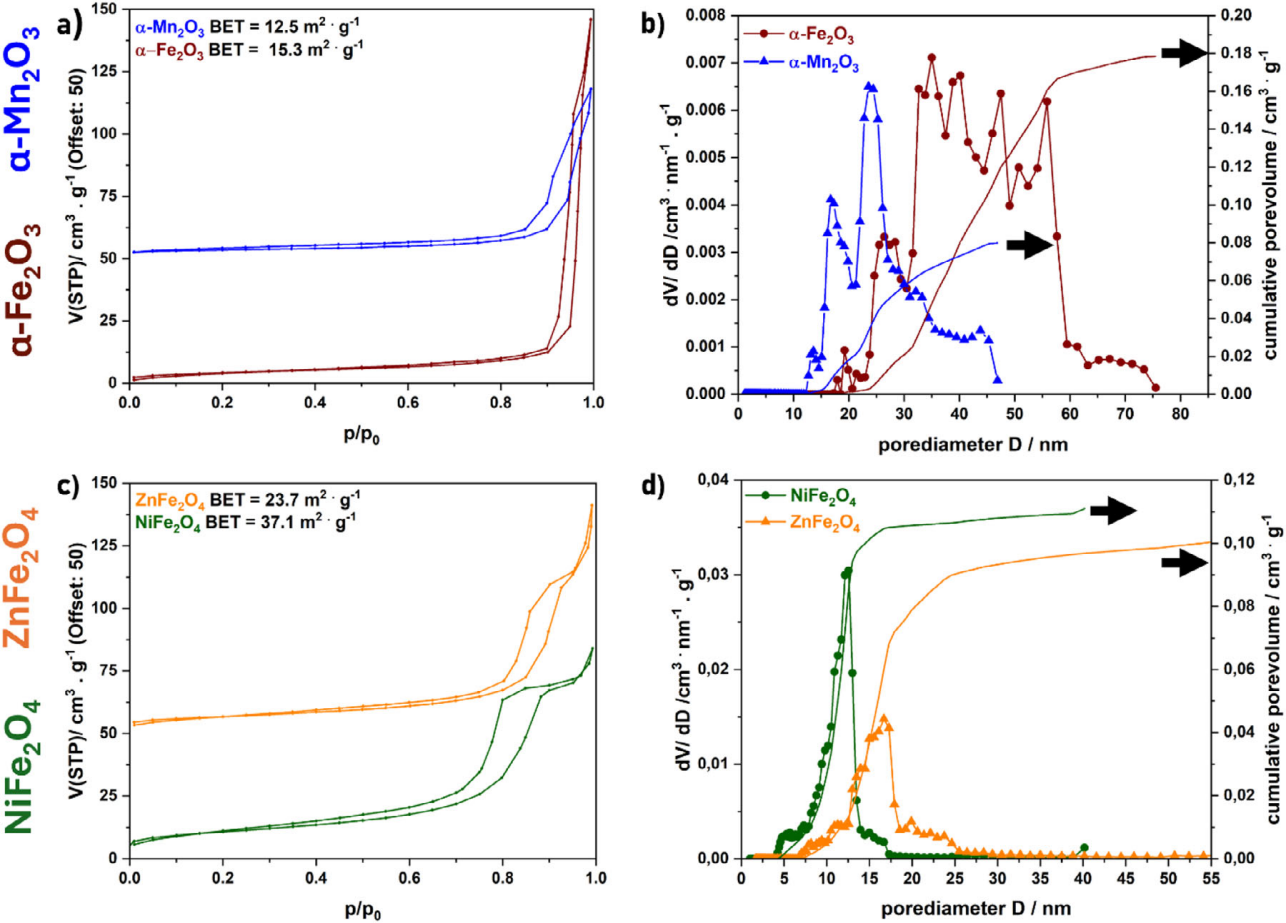
N_2_ physisorption isotherms (shifted by 50 cm^3^ g^−1^) and corresponding BET surface areas of a) α‐Fe_2_O_3_, α‐Mn_2_O_3_ and c) NiFe_2_O_4_, ZnFe_2_O_4_. Corresponding pore size distributions and cumulative pore volumes (full line) of b) α‐Fe_2_O_3_, α‐Mn_2_O_3_ and d) NiFe_2_O_4_, ZnFe_2_O_4_, calculated by NLDFT analysis of physisorption isotherms.

Nitrogen physisorption measurements were conducted to gain insights into the pore sizes of the materials. In **Figure** **3** the nitrogen physisorption isotherms (left) and corresponding pore size distribution and cumulative pore volumes (right) are presented. BET surface areas and pore volumes of the compounds are summarized in **Table** **1**.

**Table 1:** Results obtained from nitrogen physisorption measurements.

Compound	α‐Fe_2_O_3_	α‐Mn_2_O_3_	ZnFe_2_O_4_	NiFe_2_O_4_
BET surface area/ m^2^ · g^−1^	15.3	12.5	23.7	37.1
Cumulative pore volume/ cm^3^ · g^−1^	0.18	0.08	0.11	0.12

Physisorption isotherms show hystereses loops (type IV(a), IUPAC^[^ ^47^ ^]^), indicating the presence of mesopores in each sample. A type H3 isotherm is present for α‐Fe_2_O_3_ and α‐Mn_2_O_3_, whereas the ferrites show a type H5 hysteresis loop. Type H3 hystereses are often associated with non‐rigid aggregates of plate‐like particles and also macropores,^[^ ^47^ ^]^ which is in accordance with the pore size distribution obtained by nonlocal density functional theory (NLDFT) for α‐Fe_2_O_3_ and α‐Mn_2_O_3_. For α‐Fe_2_O_3_ mesopores and macropores are present in the range of 17–75 nm, with a cumulative pore volume of 0.18 cm^3^ g^−1^, while nanoparticles of α‐Fe_2_O_3_ (prepared by Gunawardhana et al.^[^ ^48^ ^]^) exhibit much smaller pore volumes. For α‐Mn_2_O_3_ pores in the range of 4–55 nm can be found, with a cumulative pore volume of 0.08 cm^3^ g^−1^. In contrast to nanoparticles prepared by Fardood et al.^[^ ^46^ ^]^ The pore size distribution is broader and also in the mesoporous regime. α‐Fe_2_O_3_ and α‐Mn_2_O_3_ prepared without P‐123 (see Figure S2c,d, Supporting Information) exhibit smaller cumulative pore volumes as well as a shift of the pore size distribution to smaller pore diameters. According to the Brunauer‐Emmet‐Teller (BET)^[^ ^49^ ^]^ model the specific surface area was calculated to be 12.5 m^2^ g^−1^ (α‐Mn_2_O_3_) and 15.3 m^2^ g^−1^ (α‐Fe_2_O_3_).

**Figure 4:**
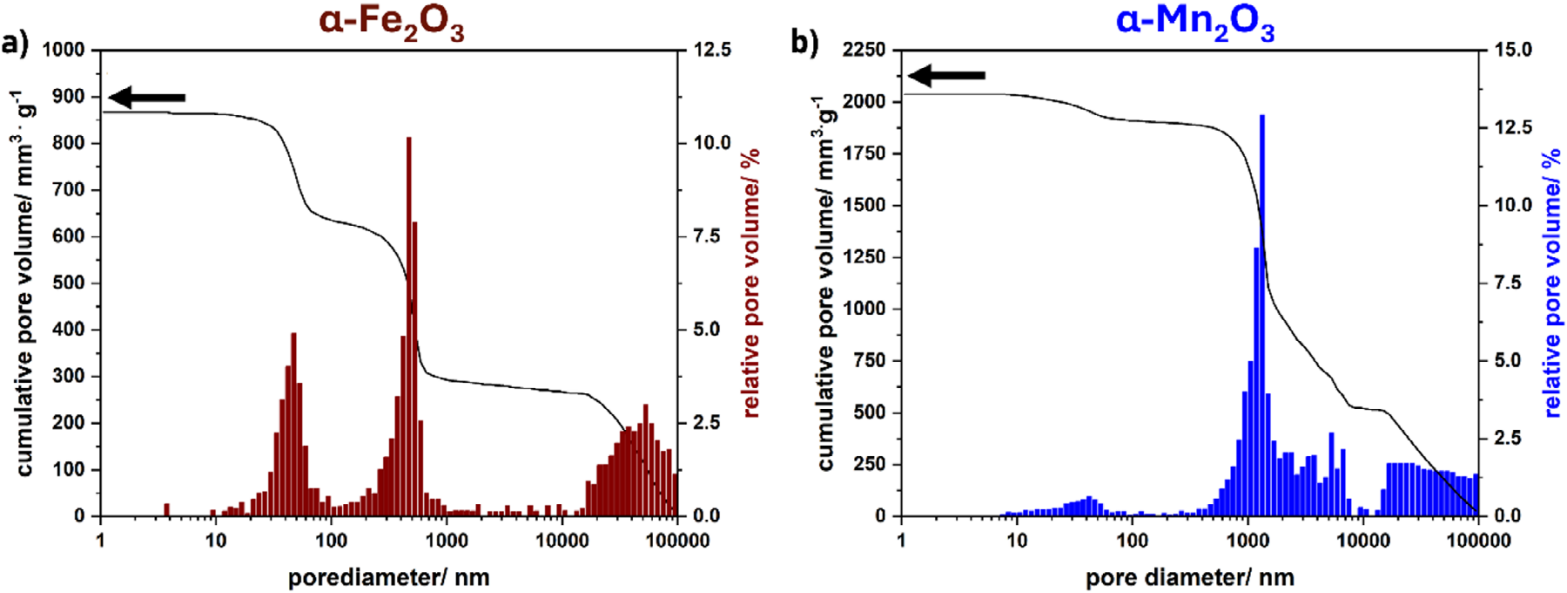
Pore size distribution and cumulative pore volumes of a) α‐Fe_2_O_3_ and b) α‐Mn_2_O_3_, determined by Mercury intrusion porosimetry.

Mercury intrusion porosimetry (MIB) (**Figure** **4**) was conducted to investigate the pore size distribution of the two latter samples in more detail, especially in the macroporous region, and is in accordance with the results obtained by nitrogen physisorption measurements for the mesoporous regime. In addition, the pore size distribution determined by MIB includes macropores up to 100 nm for both samples, and another increase in pore volume in the range from 100 nm up to 100 µm can be observed which is due to voids between the particles.

The H5 hystereses of NiFe_2_O_4_ and ZnFe_2_O_4_ (Figure 3) are associated with pore structures containing both open and partially blocked mesopores,^[^ ^47^ ^]^ which might be due to residues of P‐123. In contrast to binary metal oxides α‐Fe_2_O_3_ and α‐Mn_2_O_3_, the ferrites display a narrower pore size distribution (Figure 3d) in the mesoporous regime ranging from 5 to 18 nm for NiFe_2_O_4_ and 7 to 25 nm for ZnFe_2_O_4_, with cumulative pore volumes of 0.11 and 0.12 cm^3^ · g^−1^ respectively. The BET surface area amounts to 37.1 m^2^ g^−1^ for NiFe_2_O_4_ and 23.7 m^2^ g^−1^ for ZnFe_2_O_4_. In Figures S4 and S6 (Supporting Information) the temperature‐dependent study of NiFe_2_O_4_ and ZnFe_2_O_4_ is shown with pore size distributions of nanoparticular and mesoporous ferrites. For mesoporous ZnFe_2_O_4_ an increase in pore diameter can be observed with increasing reaction temperature. In contrast to mesoporous ferrites, the hysteresis loop of nanoparticles seems to be of type H2(a). Type H2 is associated with pore‐blocking or cavitation‐induced evaporation,^[^ ^47^ ^]^ however, this might not be an indication for mesopores, but rather for agglomerates of particles (see SEM images, Figures S4 and S6, Supporting Information) and filling of the adsorbent between voids of adjacent nanoparticles.

**Figure 5:**
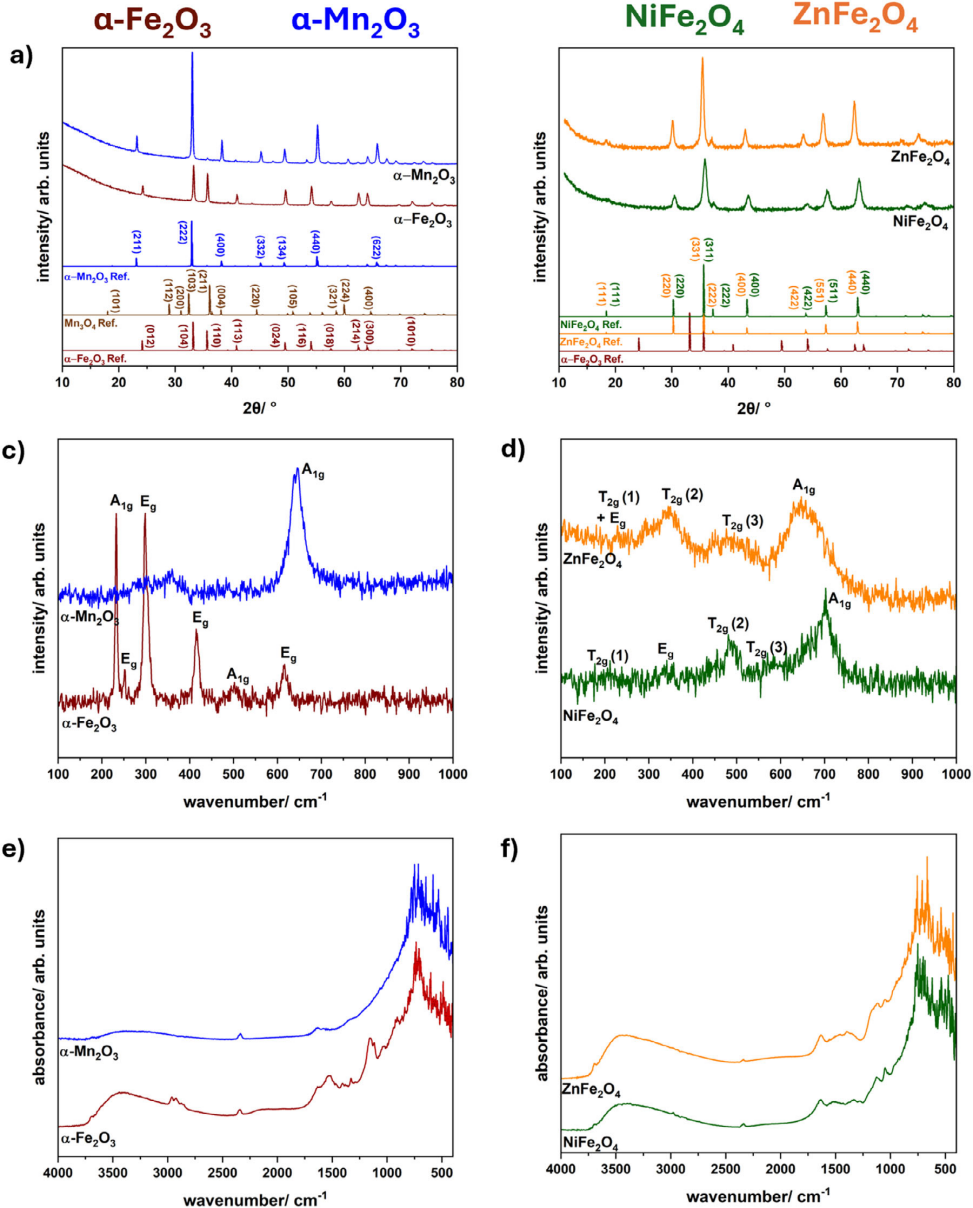
PXRD patterns (top), Raman spectra (middle) and corresponding DRIFT spectra (bottom) of a), c), e) α‐Fe_2_O_3,_ α‐Mn_2_O_3_ and b), d), f) NiFe_2_O_4_, ZnFe_2_O_4_ prepared via the microwave‐based synthesis strategy. Following COD (Crystallography Open Database) reference cards were used: α‐Fe_2_O_3_ (COD: 2 101 167), α‐Mn_2_O_3_ (COD: 1 514 103), Mn_3_O_4_ (COD: 1 011 262), NiFe_2_O_4_ (COD: 1 541 589), ZnFe_2_O_4_ (COD: 9 005 110).

Phase‐purity and crystal structure of the calcined compounds were studied by PXRD, Raman and IR spectroscopy, as shown in **Figure** **5**. Phase‐pure, crystalline compounds are obtained after the calcination step according to the PXRDs and match the diffraction and SAED pattern of the respective references. Diffraction patterns of ferrites confirm the formation of a spinel structure. No additional side phases are observed for all compounds. Rietveld refinement was performed for all PXRDs, including the PXRDs in the Supporting Information of the temperature‐dependent series, and are depicted in Figures S3, S5 and S7 (Supporting Information). The fits are in accordance with references from the literature. Even after detailed Rietveld refinement side phase formation could be excluded. Elemental mapping (Figures S8 and S10, Supporting Information) for the spinel‐type ternary ferrites shows a homogeneous distribution of all elements and no clusters or phase segregation. The metal (Ni, Zn) to Fe ratios of NiFe_2_O_4_ and ZnFe_2_O_4_ were calculated from EDXS and are close to the expected value of 0.5 (see Tables S1 and S2, Supporting Information). For some samples silicon can be detected in the EDX spectra which might be due to dissolution of silicon in the borosilicate glass of the microwave vials. High resolution XP and survey spectra are presented in Figures S12–S15 (Supporting Information). The atomic percentages of all found elements are summarized in Table S3 (Supporting Information). The presence of all used elements at the surface is confirmed in the survey scans. Traces of sodium can also be found which might be due to impurities during the synthesis procedure. The ratio of Ni to Fe amounts to 0.496, which is in good agreement to EDXS measurements. For ZnFe_2_O_4_ an undefined zinc surface species is found and the atomic ratio amounts to 0.85 (Zn:Fe). It should be noted that XPS is a surface sensitive method, therefore during synthesis zinc might have been excluded from the crystal lattice on the outside of the particles, whereas EDXS suggest that in the bulk material the ratio of Zn to Fe is in the correct magnitude. The signals for α‐Fe_2_O_3_, α‐Mn_2_O_3_ and NiFe_2_O_4_ in the high resolution spectra can be assigned according to the literature.

Since for hematite α‐Fe_2_O_3_ side phases like maghemite γ‐Fe_2_O_3_ or spinel‐type magnetite Fe_3_O_4_ might be present and undetectable by PXRD due to small quantity or amorphous nature, Raman spectroscopy was performed additionally. α‐Fe_2_O_3_ is considered to be the most stable modification under ambient conditions, however, due to high temperatures and pressure in the microwave the formation of other iron oxides cannot be excluded. Whereas the iron precursor in the microwave consists of trivalent iron cations, microwave conditions might lead to the reduction of iron cations and therefore give rise to the formation of mixed‐valent magnetite. Trivalent iron oxide maghemite on the other hand can be synthesized via the oxidation of magnetite. Subsequent thermal transformation leads to hematite.^[^ ^50^ ^]^

In Figure 5c, the Raman spectrum of hematite with the corresponding characteristic Raman modes is shown without impurities of maghemite (Reference: 500–511 cm^−1^ (E_g_), 700 cm^−1^ ($A_{1_{g}}$), Salviano et al.^[^ ^51^ ^]^) or magnetite (Reference: 306–310 cm^−1^ (E_g_), 350 –365 cm^−1^ ($T_{2_{g}}$), 450–490 cm^−1^ ($T_{2_{g}}$), 538–554 cm^−1^ ($T_{2_{g}}$), 668–672cm^−1^ ($A_{1_{g}}$), Salviano et al.^[^ ^51^ ^]^). All Raman modes at 230cm^−1^ ($A_{1_{g}}$), 250 cm^−1^ (E_g_), 300 cm^−1^ (E_g_), 415 cm^−1^ (E_g_), 500 cm^−1^ ($A_{1_{g}}$), 615 cm^−1^ (E_g_) are assigned to the internal modes, associated with motion within the octahedral units FeO_6_ of α‐Fe_2_O_3,_ and the external modes (rotations and translations of FeO_6_ units).^[^ ^52^ ^]^ Three peaks at 645, 352, and 293 cm^−1^ in the Raman spectra of α‐Mn_2_O_3_ can be assigned to spinel‐type Mn_3_O_4_ with the most intense peak at 654 cm^−1^ being the characteristic $A_{1_{g}}$ mode of spinels,^[^ ^53^ ^]^ in accordance to Julien et al.^[^ ^54^ ^]^ and Lutz et al.^[^ ^55^ ^]^ However, the diffraction pattern of the measured sample can be clearly assigned to α‐Mn_2_O_3_ as the reference pattern for Mn_3_O_4_ displays the most intense reflexes at different angles (see reference in Figure 5a). Julien et al.^[^ ^54^ ^]^ also noted that Mn_3_O_4_ is highly stable under the laser beam and Buciuman et al.^[^ ^56^ ^]^ observed changes of Raman modes in dependency of the wavelength of the laser, therefore Mn_3_O_4_ might form during spectra acquisition. Furthermore, Bernardini et al.^[^ ^57^ ^]^ characterised various MnO_x_ species by Raman spectroscopy and noted on the contradicting data in literature and concluded that the frequency of Raman modes of various manganese oxide compounds are similar.

For both ferrites, α‐Fe_2_O_3_ might be a possible side phase, as reported in the literature.^[^ ^58^ ^]^ Spinel‐type maghemite and magnetite might not be detected in the PXRDs due to similar crystal structure and diffraction pattern.^[^ ^59^ ^]^ As shown here, synthesis of α‐Fe_2_O_3_ is conducted in the same temperature range as the ferrites with the same iron precursor Fe(acac)_3_, therefore in case of non‐stoichiometric conversion of the reaction educts, α‐Fe_2_O_3_ could be formed. In addition, the inversion degree of the spinel structure can be evaluated from Raman spectroscopy if high quality data is available. A splitting in the observed modes can be attributed to the occupation of one crystal site by more than one cation. Due to the difference in mass, the change in the metal‐oxygen bond strength causes a shift of vibration frequency.^[^ ^53^ ^]^ The Raman spectrum of NiFe_2_O_4_ consist of five active Raman modes at 200 cm^−1^($T_{2_{g}}$), 330 cm^−1^ (E_g_), 480 cm^−1^($T_{2_{g}}$), 580 cm^−1^($T_{2_{g}}$) and 700 cm^−1^ ($A_{1_{g}}$),^[^ ^60^ ^]^ without any indication of the Raman modes of α‐Fe_2_O_3_ which would also display sharper peaks as shown in Figure 5c. For ZnFe_2_O_4_ five Raman modes can be allocated at 650 cm^−1^ ($A_{1_{g}}$), 480 cm^−1^($T_{2_{g}}$), 345 cm^−1^ ($T_{2_{g}}$) and at 230 cm^−1^ ($E_{g}+T_{2_{g}}$).^[^ ^51^, ^61^ ^]^ In the spinel structure the $A_{1_{g}}$ mode corresponds to the symmetric stretching modes located in tetrahedral sites whereas the other modes can be assigned to the symmetric and asymmetric stretching and bending metal‐oxygen modes in an octahedral environment.^[^ ^53^, ^62^ ^]^ A shift in frequency between NiFe_2_O_4_ and ZnFe_2_O_4_ is present due to mass differences of Zn^2+^ and Ni^2+^ cations. Furthermore, the $A_{1_{g}}$ mode of NiFe_2_O_4_ is asymmetric, suggesting a split peak and therefore an inverse spinel structure for NiFe_2_O_4_, whereas ZnFe_2_O_4_ is a normal spinel, which is in accordance with literature.^[^ ^17^, ^18^, ^51^, ^63^ ^]^

To further analyse the chemical composition of the materials, DRIFT spectra were collected. The highest intensity of these IR bands could be assigned to the metal‐oxygen vibrational modes in the range of 450–800 cm^−1^.^[^ ^64^ ^]^ Two bands are seen for the ferrites which correspond to metal‐oxygen vibrations at tetrahedral and octahedral sites.^[^ ^65^ ^]^ For α‐Mn_2_O_3_ the vibrations are less intense which might be due to the lower reflectance of the black sample. In the range of 3250–3600 cm^−1^ a broad IR band is present in all spectra, indicating the presence of lattice water. The related vibration at 1630–1600 cm^−1^ can also be found. Small IR bands can be seen above 3500 cm^−1^ which indicate the presence of individual hydroxyl groups on the surface. Signals at 1440–1410 cm^−1^ and 1620–1585 cm^−1^ can be assigned to carbonyl groups of precursor residues. Signals at 2350–2340 cm^−1^ are attributed to carbon dioxide.^[^ ^14^ ^]^ Vibrations of P‐123 are also expected in the already discussed ranges. Ether vibrations of ethylene oxide and propylene oxide are observed in the range of 1310–1020 cm^−1^ and are visible for all samples. The methyl vibration of propylene oxide is expected in the range of 1490–1150 cm^−1^. As there is an asymmetric and symmetric deformation vibration of CH_3_ two IR bands can be assigned – respectively the one at ≈1500 and 1300 cm^−1^. Another small barely visible IR band at 1400 cm^−1^ and shortly above 1000 cm^−1^ may be attributed to the deformation vibration of CH_2_ and CH. The respective C‐C‐H stretching vibrations are expected in the range of 3000 –2800 cm^−1^ and can be found for all compounds except α‐Mn_2_O_3_.^[^ ^64^, ^66^ ^]^ Nevertheless, the signal are very small overall, indicating only small amounts of residues.

**Figure 6:**
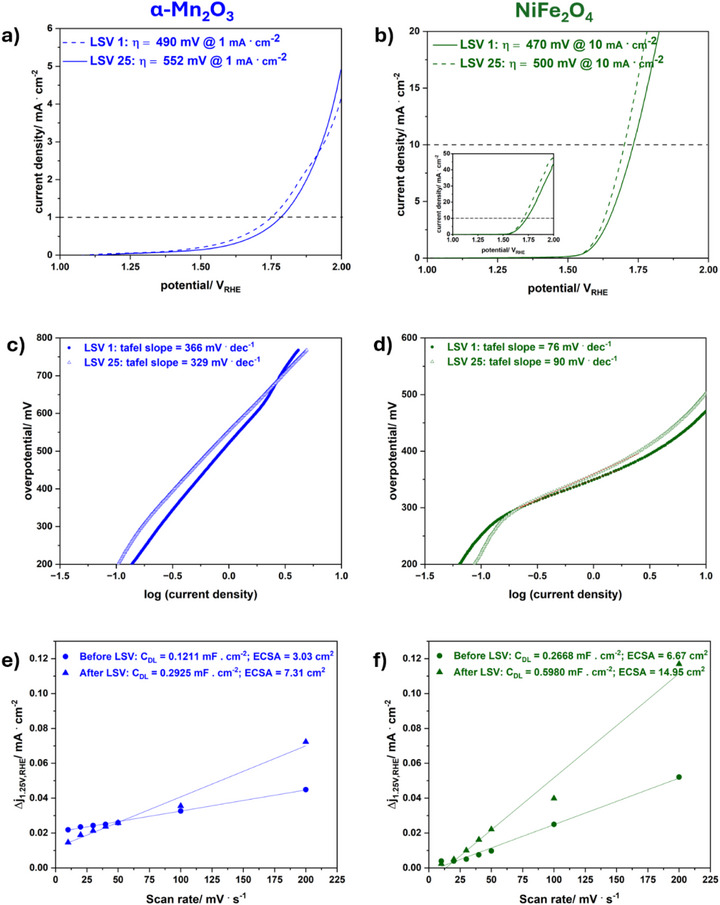
a,b) LSV scans with both the initial scan (after three previous CV scans) and the final scan, with a scan rate of 5 mV · s^−1^ in 1 m KOH, c,d) corresponding Tafel plots, e,f) ECSA slopes determined at 1.25 V before and after LSV as a function of the scan rate of α‐Mn_2_O_3_ (left) and NiFe_2_O_4_ (right).

To investigate the functional advantages of the mesoporous materials, NiFe_2_O_4_ and α‐Mn_2_O_3_ were tested for electrocatalytic OER. In **Figure** **6** electrochemical investigations of NiFe_2_O_4_ and α‐Mn_2_O_3_ are presented. Experiments were conducted under alkaline conditions (1 m KOH) using a three‐electrode system. Carbon paper with respectively 3.0 mg · cm^−2^ of NiFe_2_O_4_ and α‐Mn_2_O_3_ were used as the working electrode. Parameters obtained from electrochemical measurements are summarized in **Table** **2**. Differences of forward and backward scans at 1.25 V vs RHE (reversible hydrogen electrode) were plotted against the scan rate, as shown in Figure 6 e,f. The linear slope represents the double layer capacitance C_DL_. By division with the specific capacitance (typical value of 0.040 mF for oxidic materials) the electrochemical active surface area (ECSA) is obtained. A standard value for the specific capacitance is typically used for evaluation of the electrocatalyst, which is reported to lead to a variation of the ECSA by a factor as much as seven, according to McCrory et al. By the preparation of an atomically smooth planar surface, the specific capacitance could be determined for the materials in this work, which is not possible due to the spherical morphology of the mesoporous compounds. Additional contributions such as pseudo‐capacitance from ion adsorption or intercalation, and chemical capacitance arising from electron trap states are neglected and the method also assumes that all metal oxide catalysts possess similar electrical conductivity. Therefore, the value of the ECSA needs to viewed with careful consideration.^[^ ^6^ ^]^ Cyclic voltammetry (CV) scans for ECSA calculation with varying scan rate before and after linear sweep voltammetry (LSV) are shown in Figure S16 (Supporting Information). The values determined for a scan rate of 5 mV s^−1^ were neglected during linear fitting.

**Table 2:** Parameters obtained from electrochemical investigations on NiFe_2_O_4_ and α‐Mn_2_O_3_. The overpotential for OER vs RHE was determined at a current density of 1 mA · cm^−2^ (α‐Mn_2_O_3_) and 10 mA · cm^−2^ (NiFe_2_O_4_).

	η/mV vs RHE [after LSV]	Tafel slope/mV · dec^−1^ [after LSV]	ECSA/cm^2^ [before LSV]	ECSA/cm^2^ [after LSV]
NiFe_2_O_4_	500	90	6.67	14.95
α‐Mn_2_O_3_	552	329	3.03	7.31

Electrochemical impedance spectroscopy (EIS) (Figure S17a,b, Supporting Information) was employed to determine the resistance of the electrode before and after LSV. Due to electrochemical activation of the electrode during LSV sweeps and removal of organic residues, the resistance might change. The system resistance of the experimental setup was measured before and after measurement and amounts to ≈3 Ω. For NiFe_2_O_4_ the resistance decreases from ≈200 Ω to a low resistance of ≈15 Ω after LSV, indicating a fast charge transfer and activation of the electrode during LSV sweeps. This is in accordance with the strong increase of the ECSA from 6.67 to 14.95 cm^2^. In contrast, the resistance of α‐Mn_2_O_3_ on carbon paper before and after LSV is ≈350 Ω. An increase in the ECSA can be observed but the difference before and after LSV sweeps is smaller than for NiFe_2_O_4_. By LSV the overpotential at 10 mA · cm^−2^ for NiFe_2_O_4_ was determined to be 500 mV vs RHE. The current density reaches values up to 40 mA · cm^−2^. For α‐Mn_2_O_3_ an overpotential of 552 mV vs RHE was determined at a current density of 1 mA · cm^−2^. The Tafel slopes amount to 90 mV · dec^−1^ (NiFe_2_O_4_) and 329 mV · dec^−1^ (α‐Mn_2_O_3_) respectively. The low Tafel slope for NiFe_2_O_4_ is in accordance with reported Tafel slopes for spinel‐type electrocatalysts.^[^ ^13^, ^67^ ^]^ CV scans were conducted before and after LSV and are shown in Figure S17c,d (Supporting Information). Activity can be measured in the OER regime. Other redox features are also visible which might indicate changes of the catalyst and valency for Mn(III) and Ni(II) during OER. For NiFe_2_O_4_ the redox pairs Ni^2+^/Ni^3+^ and Fe^2+^/Fe^3 +^ are present. The redox feature indicates an irreversible reduction, possibly of Fe^3 +^ to Fe^2+^. The peak between 0 and 0.2 V can be assigned to the Fe^2+^ to Fe^3+^ transition, as reported by Volk et al.^[^ ^68^ ^]^ Mn(III) is known for changing valency during CV and also disproportion of Mn(III) to Mn(II)/ Mn(IV) is possible.^[^ ^69^, ^70^ ^]^ Therefore, the redox feature below 0.5 V is associated with irreversible changes in valency of manganese during OER. A 24 h chronopotentiometric long‐term stability test was performed for NiFe_2_O_4_ at 10 mA · cm^−2^ and is presented in Figure S18 (Supporting Information). First, there is a significant decrease in the voltage (and therefore overpotential) which might be attributed to the electrochemical activation of the electrode. After a slight increase in overpotential at ≈10 h a stable potential level of 1.8 V (overpotential of 570 mV vs RHE) is observed. The increase in overpotential can be attributed to the degradation of the carbon paper electrode. Fine brown particles were observed in the electrolyte after the long‐term measurement. After the OER and chronopotentiometric measurements of NiFe_2_O_4_ the electrolyte was tested for Fe^2+^, Fe^3+^, Ni^2+^ ions with respectively K_4_[Fe(CN)_6_], K_3_[Fe(CN)_6_] and dimethylglyoxime, as known in literature^[^ ^71^ ^]^ (see Figure S19, Supporting Information) and characterized by UV–Vis spectroscopy (Figure S20, Supporting Information). The ions could not be detected by precipitation, nor were the d‐d transitions in the visible spectrum of light observable in the UV–Vis spectra. However, an increase of intensity down to 300 nm is detected. The *π*–*π* ^*^ transitions of carbon are expected in the range from 180 to 260 nm, therefore the increase in the UV range confirms that the carbon paper is degraded and dissolved in the electrolyte during measurement.^[^ ^72^ ^]^

Simon et al. prepared nanoparticles^[^ ^14^ ^]^ of NiFe_2_O_4_ as well as mesoporous^[^ ^13^ ^]^ NiFe_2_O_4_ by the EISA process. An increase in overpotentials with increasing calcination temperature was determined for the compounds. Nanoparticles calcined at 500 °C exhibited an overpotential of 539 mV vs RHE whereas mesoporous NiFe_2_O_4_ by EISA demonstrated an overpotential greater than 570 mV when calcined at 550 °C. According to Simon et al.^[^ ^13^ ^]^ The loss in activity of mesoporous NiFe_2_O_4_ is due to pore shrinkage or pore collapse during crystallite growth of the mesoporous material. Furthermore, calcination might lead to smaller surface areas^[^ ^18^ ^]^ and therefore less active sites. The pore size distributions of microwave‐ (this work) and EISA‐derived mesoporous NiFe_2_O_4_ calcined at 550 °C are in the same range. Although similar pore size distribution, mesoporous NiFe_2_O_4_ prepared via microwave route exhibits a lower overpotential of 500 mV vs RHE. NiFe_2_O_4_ presented in this work also reaches higher current densities in comparison to NiFe_2_O_4_ nanoparticles and mesoporous NiFe_2_O_4_ calcined at 500 °C and higher by Simon et al.

The spherical, sponge‐like morphology of microwave mesoporous NiFe_2_O_4_ seems to be more favourable for electrocatalytic performance, which might be due to improved charge carrier transport through the nanostructured material. Although, the BET surface areas obtained for the nanoparticles (63 m^2^ · g^−1^ @ 500 °C^[^ ^14^ ^]^) as well as for mesoporous NiFe_2_O_4_ (44 m^2^ · g^−1^ @ 550 °C^[^ ^13^ ^]^) by EISA process are higher than for microwave‐prepared NiFe_2_O_4_ presented in this work (37 m^2^ · g^−1^ @ 550 °C), the size of the ECSA does not correlate with the BET surface areas, with an ECSA of 0.62 cm^2^ (C_DL_ = 0.26 mF · cm^−2^) for NiFe_2_O_4_ nanoparticles, 18 cm^2^ for mesoporous NiFe_2_O_4_ (EISA) by Simon et al. (C_DL_ = 0.73 mF · cm^−2^) and this work (14.95 cm^2^) (C_DL_ = 0.60 mF · cm^−2^). However, the mass of the electrocatalyst for evaluation of the ECSA also needs to be considered. For loading of carbon paper with microwave‐prepared NiFe_2_O_4_ 3 mg were used whereas glassy carbon electrodes (Simon et al.) were loaded with half the amount of mesoporous electrocatalyst and one tenth of nanoparticles respectively. In relation to each other, microwave‐derived mesoporous NiFe_2_O_4_ still exhibits a higher ECSA than the respective nanoparticular compounds. It should also be noted that in contrast to Simon et al. who used a glassy carbon electrode, carbon paper was employed here as electrode because it is not OER active. Zander et al.^[^ ^18^ ^]^ presented a comparative study of NiFe_2_O_4_ on glassy carbon, carbon paper and nickel foam and found lower activity when measured on carbon paper. Literature reports on lower overpotentials^[^ ^73^, ^74^, ^75^ ^]^ for NiFe_2_O_4_ are also available, however in the present work emphasis is placed on the short reaction times provided by microwave synthesis as well as the spherical morphology which seems to display improved activity in comparison to similar materials. The loss in activity with increasing calcination temperature, and therefore increasing crystallinity, was not observed for the material presented in this work.

α‐Mn_2_O_3_ is capable of OER due to the Jahn–Teller distortion of edge sharing octahedron which are occupied by Mn^3+^. Due to the distortion the metal oxide bond is elongated, thus reducing bond energy which enables the compound to take part in OER. Mn(III) is considered to be an essential intermediate state for achieving catalytic reactions.^[^ ^76^, ^77^ ^]^ Nanostructuring of the electrocatalyst increases the BET surface area as well as the ECSA, which gives rise to more Mn(III) active sites. The overpotential of prepared α‐Mn_2_O_3_ is 552 mV vs RHE at a current density of 1 mA · cm^−2^ with an ECSA of 7.31 cm^2^. Mesoporous α‐Mn_2_O_3_ is electrochemical active with current densities of α‐Mn_2_O_3_@carbon paper falling below values reported in literature. As already mentioned, activity might be lower on carbon paper. Ramírez et al.^[^ ^78^ ^]^ determined an overpotential of 570 mV vs RHE at a current density of 20 mA · cm^−2^ in 1 m KOH for films of α‐Mn_2_O_3_ whereas Gorlin and Jaramillo^[^ ^8^ ^]^ also achieved high current densities at a potential of 1.77 V vs RHE.

In summary, microwave‐derived mesoporous NiFe_2_O_4_ exhibits a lower overpotential of 500 mV and a higher ECSA of 14.95 cm^2^ than nanoparticles of NiFe_2_O_4_ calcined at 500 °C, and a lower overpotential in contrast to mesoporous NiFe_2_O_4_ prepared by the EISA process. The spherical morphology might be more favourable for OER. A long‐term stability test showed stable voltage output, making the compound favourable for applications.

## 3 Conclusion

We have presented a novel quick and facile microwave synthesis method for the preparation of mesoporous binary and ternary metal oxides (α‐Fe_2_O_3_, α‐Mn_2_O_3_, NiFe_2_O_4_, ZnFe_2_O_4_) with the porogen P‐123. After only 15–30 min of synthesis, a calcination step is necessary to remove organic residues of the precursor molecules and the porogen P‐123, as well as to increase crystallinity. Spherical, sponge‐like mesoporous metal oxides with BET surface areas ranging from 12 to 37 m^2^ · g^−1^ and cumulative pore volumes of 0.08 to 0.18 cm^3^ · g^−1^ were obtained. α‐Mn_2_O_3_ and NiFe_2_O_4_ were employed as electrocatalysts for alkaline OER. Both latter compounds are electrochemically active, with NiFe_2_O_4_ reaching current densities of up to 40 mA · cm^−2^ and an overpotential of 500 mV vs RHE at 10 mA · cm^−2^. Interestingly, NiFe_2_O_4_ exhibits a lower overpotential than comparable nanostructured materials calcined at the same temperature which could be attributed to the porous structure of the particles. The synthesis procedure reported here paves the way for other complex metal oxides to be easily prepared in fast fashion with mesoporous morphology.

## 4 Experimental Section

Chemicals were purchased from commercial providers and used without further purification. Ultrapure water was obtained by the ultrapure water system A32 by VWR. Iron(III)acetylacetonate was obtained from ACROS ORGANICS with a purity of >99%. Nickel(II)acetylacetonate was obtained from Merck KGaA. Zinc(II)acetate dihydrate was purchased from Alfa Aesar GmbH & Co. KG with a purity of >97%. Manganese(III)acetylacetonate was acquired from Tokyo Chemical Industry with a purity greater than 98%. 1‐Phenylethanol was also purchased from Sigma–Aldrich (purity: 98%) as well as Pluronic P‐123 (P‐123 (ethylene oxide)_20_(propylene oxide)_70_(ethylene oxide)_20_) with an average molecular weight of 5.800 g mol^−1^. Dimethylglyoxime was obtained from Glentham Life Sciences with a purity greater than 99%. Potassium hexacyanoferrate(II) trihydrate was obtained from Grüssing GmbH with a purity of 99% and potassium hexacyanoferrate(III) from ROTH with a purity greater than 99%. N‐pentane and diethylether (≥ 99.5%) were purchased from Brenntag and Staub & Co‐Silbermann respectively. 2‐Propanol (99.5%) was purchased from VWR Chemicals BDH with a purity greater than 99.5%. Nafion (5% w w^−1^ in water and 1‐propanol) was obtained by Alfa Aesar GmbH & Co. KG. For the preparation of the TEM samples LiChrosolv ethanol was obtained from Merck with a purity greater than 99.9%. KOH pellets were purchased from ROTH with a purity of >85% and ultrapure water was used for preparation of the electrolyte.

### Synthesis

Microwave synthesis was performed in 30 ml glass vials in an Anton Paar Monowave 400 laboratory microwave operating at 2.45 GHz. The precursor metal acetylacetonates (metal acetate dihydrate for zinc) were dissolved in 10 ml 1‐PE at RT whereas 20 mg of the porogen Pluronic P‐123 were dissolved in 5 ml 1‐PE at 40 °C for 30 min. For binary compounds α‐Fe_2_O_3_ and α‐Mn_2_O_3_ 1.5 mmol of the respective precursor was used and for ternary compounds NiFe_2_O_4_ and ZnFe_2_O_4_ 1 mmol of the iron precursor and 0.5 mmol of the respective metal precursor were used. Then, the solutions were united, stirred for 20 min at 40 °C and transferred to a glass vial and immediately processed. The vial was sealed and placed in the microwave, where it was heated to the reaction temperature as fast as possible under stirring at 600 rpm (α‐Mn_2_O_3_: 225 °C, α‐Fe_2_O_3_: 250 °C, NiFe_2_O_4_: 240°, ZnFe_2_O_4_: 275 °C). Pressures ≈2–4 bar were reached. After a set reaction time (α‐Mn_2_O_3_: 15 min, α‐Fe_2_O_3_: 30 min, NiFe_2_O_4_: 30 min, ZnFe_2_O_4:_ 15 min), the solution was cooled with compressed air to 55 °C. 25 ml n‐pentane were added to sediment the particles and the solution was cooled for 10 min in the freezer and afterward centrifuged at RT and 9000 rpm for 2 min. Then, the solid was dissolved in diethyl ether and again centrifuged at RT and 9000 rpm for 2 min. The residues of α‐Fe_2_O_3_, α‐Mn_2_O_3_ and NiFe_2_O_4_ were dried overnight at 80 °C and ZnFe_2_O_4_ at RT. The samples were calcined at 550 °C for 5 h (Heating rate: 1 K min^−1^).

### Characterization

PXRDs of NiFe_2_O_4_, ZnFe_2_O_4_, α‐Fe_2_O_3_ (without P‐123) and α‐Mn_2_O_3_ (without P‐123) were measured on an Empyrean X‐ray diffractometer by Malvern PANalytical using a CuKα source (λ = 1.541 Å) and a PIXcel 3D detector. A spinning sample holder in Bragg‐Brentano geometry was used. The lower and upper level for pulse‐height discrimination (PHD) values were set to 8.05 and 11.27 keV on the detector for samples containing iron to eliminate the fluorescence background. PXRDs of α‐Fe_2_O_3_ (with P‐123) and α‐Mn_2_O_3_ (with P‐123) were measured on a SmartLab von Rigaku SE, with a CuKα rotating anode. Rietveld Refinement was performed with the software GetControl and FullProf. Structural parameters reported in literature were used for refinement (COD: 2 101 167 (α‐Fe_2_O_3_)), (COD: 1 514 103 (α‐Mn_2_O_3_)), (COD: 2 300 289 (NiFe_2_O_4_)), (COD: 9 005 110 (ZnFe_2_O_4_)). Refined parameters include instrumental zero shift, background, FWHM (Full Width Half Maximum) parameters X and Y, lattice parameters, isotropic B, scale, size anisotropy and occupation.

DRIFT spectra were acquired with a Bruker alpha II. Raman measurements were conducted on a Horiba Yvon Raman microscope, equipped with a 11.5 W He‐Ne laser (*λ* = 633 nm). The laser intensity was reduced by using filters. A D 0.6 filter for the ferrites and hematite and a D 0.3 filter for manganese oxide was used. The exposure time was set to 20 s and the accumulation number was set to 2. Calibration was performed with a silicon wafer as reference before measurements. UV–Vis spectra of the electrolyte were recorded on a PerkinElmer Lambda 750 UV/Vis–NIR spectrometer in a wavelength range of 800–350 nm and a step size of 1 nm.

Nitrogen physisorption measurements were carried out at 77 K using an ASiQ‐MP‐MP‐AG instrument (Anton Paar QuantaTec, Boynton Beach, USA). Samples were outgassed in a vacuum at 120 °C for 12 h prior to the measurement. Data analysis was performed using ASiQwin software (Anton Paar QuantaTec, Boynton Beach, USA). The analysis of the BET surface area was carried out in the range between p/p_0_ = 0.05–0.3. Mercury intrusion porosimetry was performed by a Pascal 140/440 porosimeter from ThermoFisher Scientific. Measurements were carried out in a pressure range of 0–400 MPa. A contact angle of 140° and surface tension of 0.48 N m^−1^ were assumed for mercury. Data analysis was performed using the software Sol.I.D and the pore sizes were calculated according to Washburn equation.

XPS was performed on a Physical Electronic (PHI) VersaProbe III scanning XPS microprobe instrument equipped with a monochromatized Al Kα source. The beam voltage was set to 15 kV, the X‐Ray power was set to 50 W and a beam diameter of 200 µm was used. Survey scans were recorded with a step size of 0.4 eV, a step time of 50 ms at a pass energy of 224 eV, in the range of 1200–0 eV, two cycles. High resolution spectra were recorded with a step size of 0.1 eV, a step time of 50 ms, at a pass energy of 26 eV, 15 cycles (30 cycles for C 1*s*). To minimize charging effects, the samples were continuously flooded with both electrons and Argon ions at low energy. XPS data were analyzed with CasaXPS employing Shirley‐type backgrounds.^[^ ^79^ ^]^ Peak fitting was performed using a mixed Gaussian–Lorentzian (GL(30)) line shape, comprising 70% Gaussian and 30% Lorentzian character. All spectra were charge‐corrected by setting the C–C component of the C 1*s* peak to 284.8 eV. Curve fitting of the adventitious carbon signal followed the fitting parameters reported by Biesinger et al.^[^ ^80^ ^]^ Fe 2*p*, Mn 2*p*, Ni 2*p*, and O 1*s* signals were fitted based on parameters reported by Biesinger et al.^[^ ^81^ ^]^ Percentages of total area and spectral component separations were fixed with regard to the first component (lowest binding energy) of the signal. Due to a higher pass energy of 26 eV, values of the full width at half maximum (FWHM) of the individual components were allowed to be slightly larger than reported by Biesinger et al.^[^ ^81^ ^]^ The Fe 2*p* signal of ZnFe_2_O_4_ was deconvoluted based on the peak shapes developed by Biesinger et al. for NiFe_2_O_4._^[^ ^81^ ^]^ A shift of the first component to a higher binding energy was observed as reported earlier.^[^ ^82^ ^]^

SEM and EDXS were performed by a Zeiss Leo 1530 device, equipped with an ultra‐dry EDXS detector at an acceleration voltage of 3 and 20 kV, respectively, after sputter coating with platinum (Cressington Sputter Coater 208 HR). The EDX was calibrated with a copper disk standard and the peaks were assigned via the software Pathfinder. TEM was performed on a 200 kV JEOL JEM‐2200FS EFTEM, equipped with a Schottky FEG and an omega in‐column energy filter. Samples were dispersed in ethanol via ultrasonification and placed on a copper grid which was impregnated with carbon.

For the preparation of the electrodes 10 mg of the sample, 300 µL isopropyl alcohol and 20 µL Nafion were mixed and treated in the ultrasonic bath for 20 min. Then, respectively 25 µL of the dispersion were dripped three times on carbon paper (GDL E20/H23 from Freudenberg). A 1.0 cm × 1.0 cm area was created by covering the rest of the carbon paper with Kapton tape. The electrodes were dried overnight at RT and weighed. A three‐electrode setup was used for electrochemical measurements with 1 m KOH as electrolyte that was continuously purged with Argon 5.0. The carbon paper was employed as working electrode, and a standard reversible hydrogen reference electrode (Gaskatel) was used as a reference electrode. The counter electrode was made of a platinum net, and a Selemion AMV‐N anion exchange membrane (AGC group) was employed. The cell was connected to a Gamry Reference 3000 potentiostat and the software Gamry Frameworks was used. Before and after the measurement the system resistance was measured. EIS measurements were conducted at 1.7 V. LSV sweeps were conducted in a range of 1.0 to 2.0 V with a scan rate of 5 mV s^−1^. CVs were measured in a range of −0.7–2.0 V with a scan rate of 20 mV s^−1^, three circles each. For determination of the ECSA CV scans in the range of 1.2– 1.3 V with a scan rate of respectively 5, 10, 20, 30, 40, 50, 100, 200 mV s^−1^ were made.

## Conflict of Interest

The authors declare no conflict of interest.

## Author Contributions

J.H. contributed to the conceptualization, formal analysis, investigation, methodology, validation, visualization, and writing of the original draft. J.T. contributed to the conceptualization, formal analysis, investigation, methodology, and writing – review and editing. L.S. performed and evaluated the XPS analysis, and R.M. contributed to the conceptualization, data curation, funding acquisition, methodology, project administration, resources, supervision, and writing – review and editing.

## Supporting information



Supporting Information

## Data Availability

The data that support the findings of this study are available from the corresponding author upon reasonable request.
