# Supplementary material for: Polymer‐Assisted Direct and Rapid Microwave Synthesis of Mesoporous Binary and Ternary Metal Oxides for Electrocatalytic Water Oxidation
Source: Small. 2025 Dec 17;22(8):e10771. doi: 10.1002/smll.202510771 (PMC12877997; doi:10.1002/smll.202510771)
Supplement: Supplementary file 1 — Supporting Information [file SMLL-22-e10771-s001.pdf]

NANO · MICRO
small

Supporting Information

for *Small*, DOI 10.1002/smll.202510771

Polymer-Assisted Direct and Rapid Microwave Synthesis of Mesoporous Binary and Ternary Metal Oxides for Electrocatalytic Water Oxidation

*Jasmin Helgert, Jana Timm, Lion Schumacher and Roland Marschall**

Supporting Information

Table S1. Atomic weight percentages of elements in $NiFe_2O_4$ samples, determined by EDXS.

T/ °C	225°C Nanoparticles	225°C	230°C	235°C	240°C	245°C	250°C
Ni/ at%	10.1	11.0	10.2	7.7	6.6	12.3	10.5
Fe/ at%	22.7	24.3	22.2	15.6	14.0	25.8	24.3
Ni:Fe	1:2.25	1:2.21	1:2.17	1:2.03	1:2.12	1:2.10	1:2.31

Table S2. Atomic weight percentages of elements in $ZnFe_2O_4$ samples, determined by EDXS.

T/ °C	225°C	235°C	245°C	250°C	255°C	265°C	270°C	275°C	275°C Nanoparticles
Zn/ at%	12.1	8.1	12.6	12.3	13.1	14.5	13.3	10.3	12.4
Fe/ at%	24.8	16.2	25.4	25.7	27.5	29.6	26.5	21.0	25.1
Zn:Fe	1:2.05	1:2	1:2.02	1:2.09	1:2.10	1:2.04	1:1.99	1:2.04	1:2.02

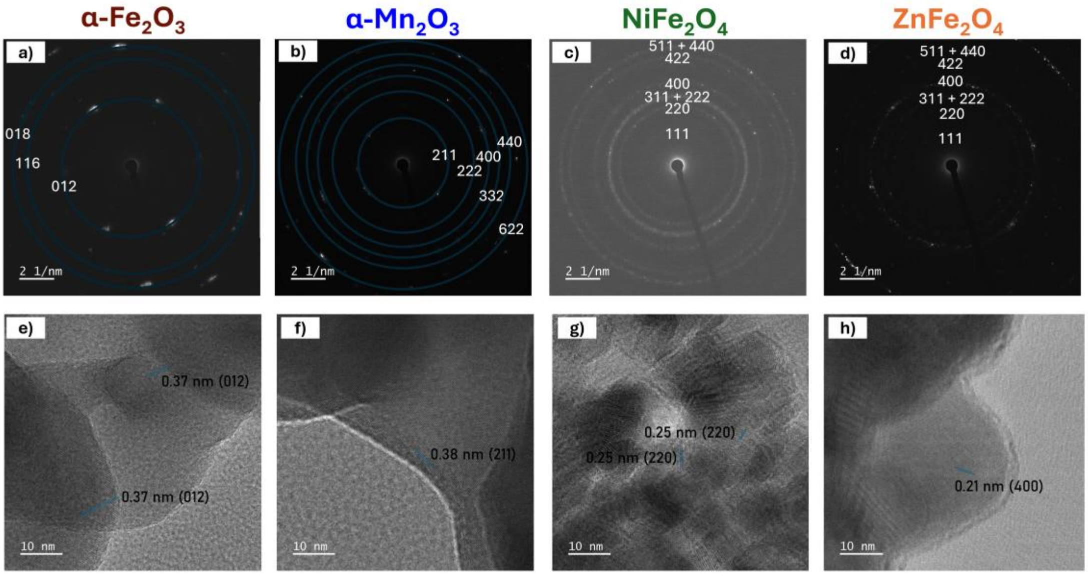

Figure S1. SAED patterns (top) and TEM images for determination of the d-spacing (bottom) of porous a), e) α-Fe_2O_3, b), f) α-Mn_2O_3, c), g) $NiFe_2O_4$ and d), h) $ZnFe_2O_4$.

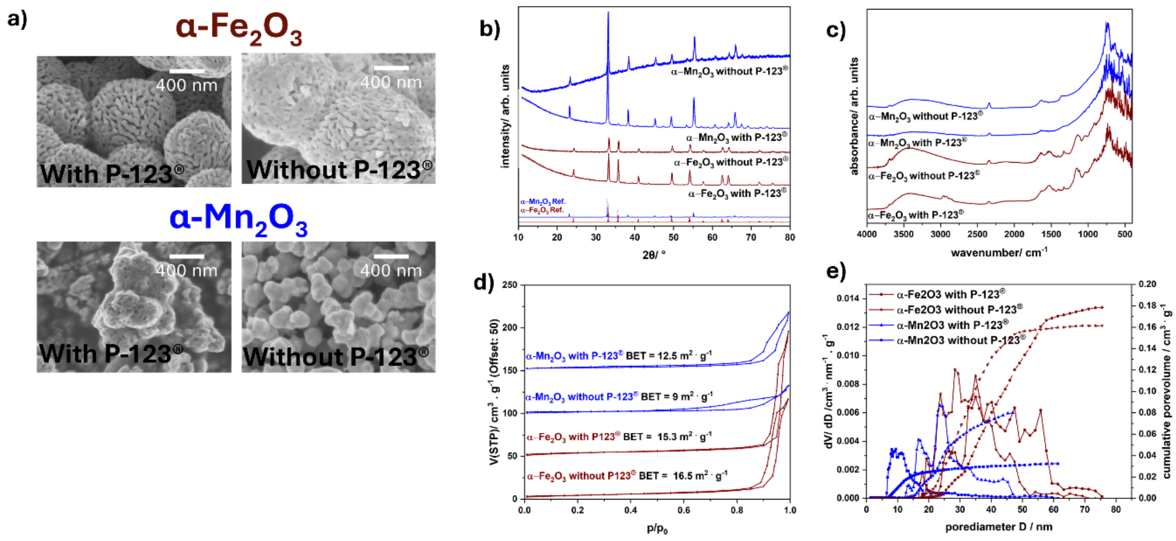

Figure S2. a) SEM images of α-Fe_2O_3 and α-Mn_2O_3 samples with and without P-123®, b) corresponding PXRDs, c) DRIFT spectra, d) physisorption isotherms (shifted by 50 $cm^3 \cdot g^{-1}$) and e) pore size distributions. PXRDs of samples with and without P-123® were measured by different diffractometers. Following COD reference cards were used: α-Fe_2O_3 (COD: 2101167), α-Mn_2O_3 (COD: 1514103).

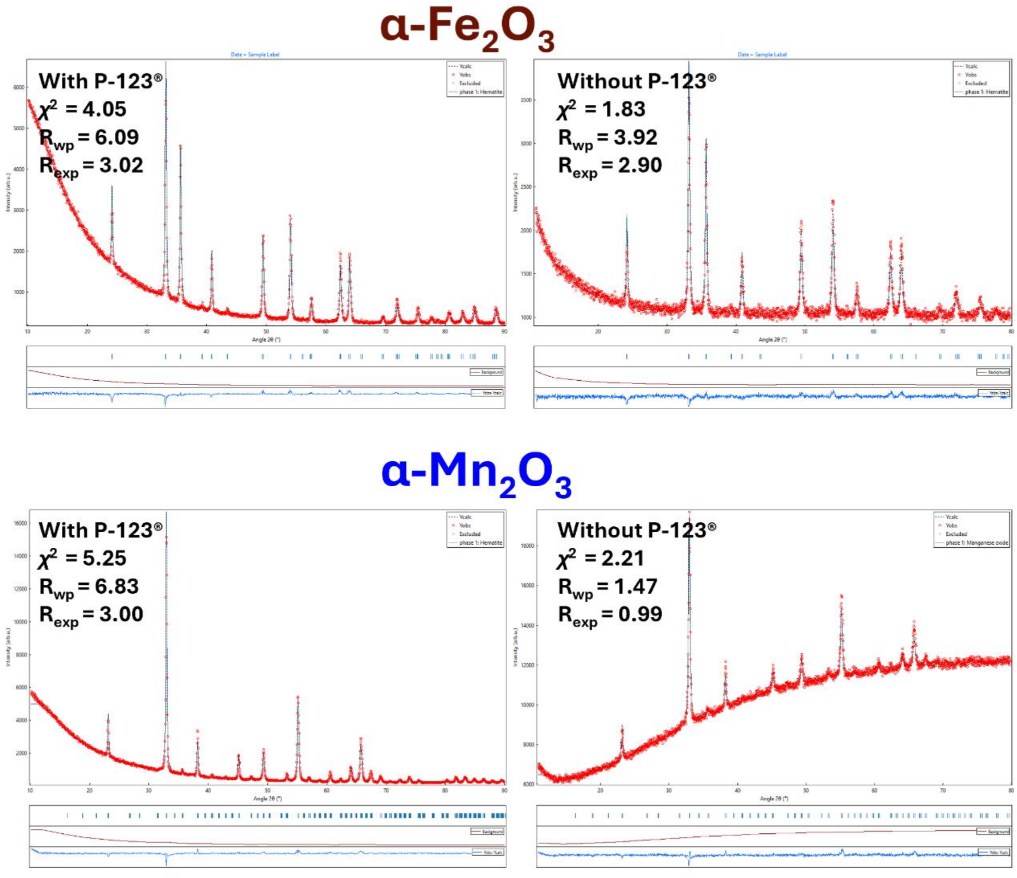

Figure S3. Rietveld refinement results of α-Fe_2O_3 and α-Mn_2O_3 with and without P-123 ®.

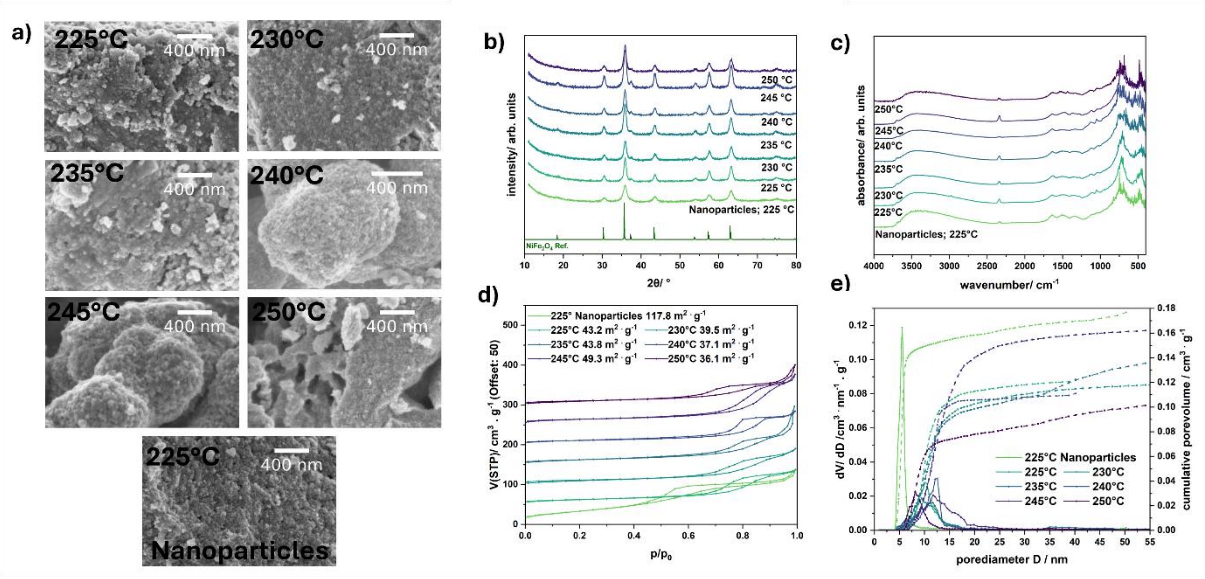

Figure S4. a) SEM images of mesoporous $NiFe_2O_4$ samples and nanoparticles synthesized at different temperatures, b) corresponding PXRDs, c) DRIFT spectra, d) physisorption isotherms (shifted by 50 $cm^3 \cdot g^{-1}$) and e) pore size distributions. Following COD reference card was used: $NiFe_2O_4$ (COD: 1541589).

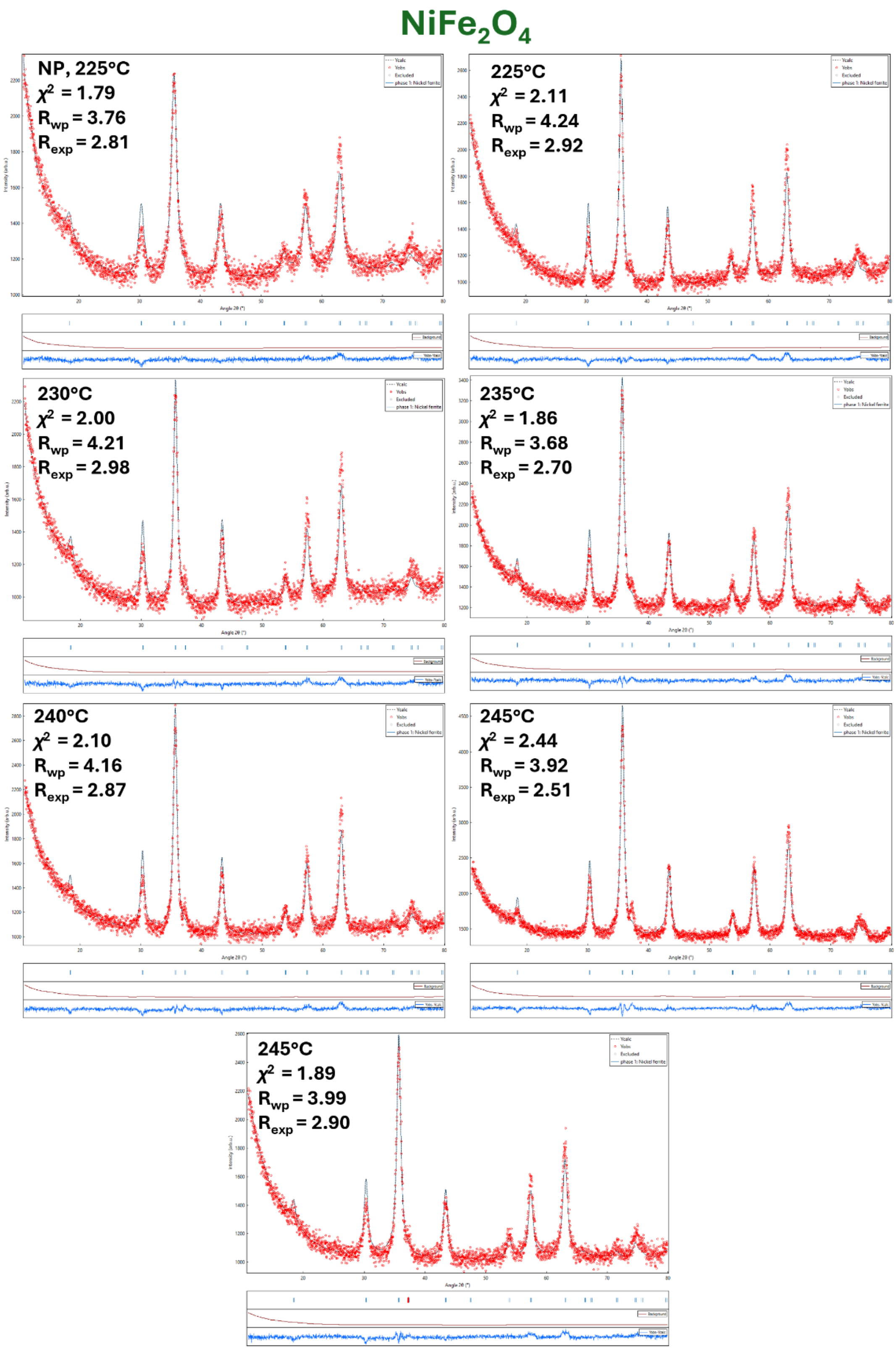

Figure S5. Rietveld refinement results of $NiFe_2O_4$.

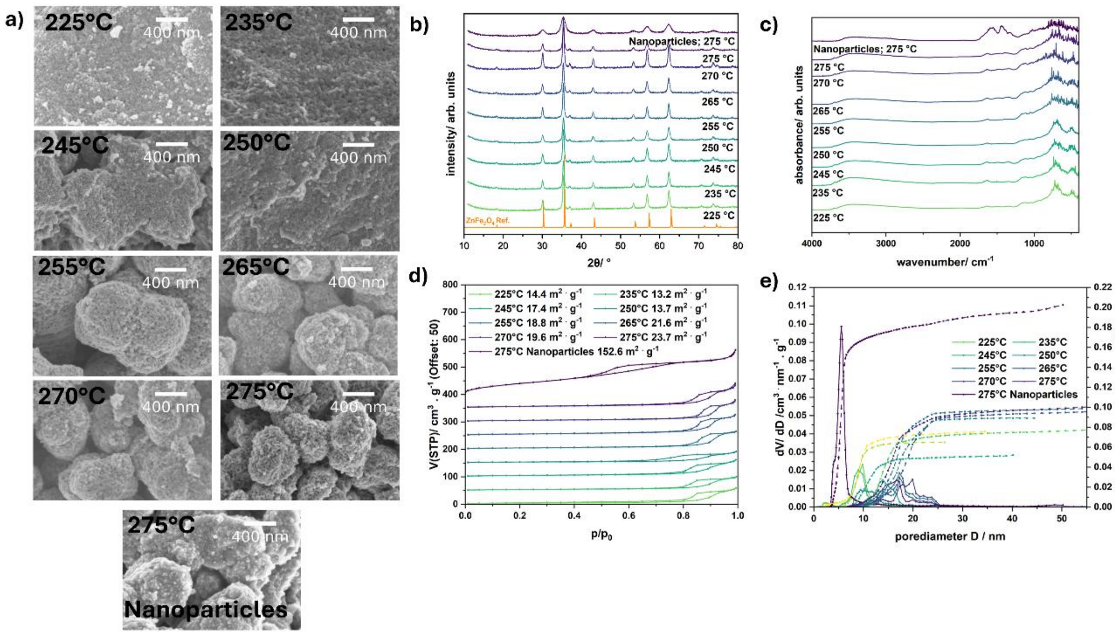

Figure S6. a) SEM images of mesoporous $ZnFe_2O_4$ samples and nanoparticles synthesized at different temperatures, b) corresponding PXRDs, c) DRIFT spectra, d) physisorption isotherms (shifted by 50 $cm^3 \cdot g^{-1}$) and e) pore size distributions. Following COD reference card was used: $ZnFe_2O_4$ (COD: 9005110).

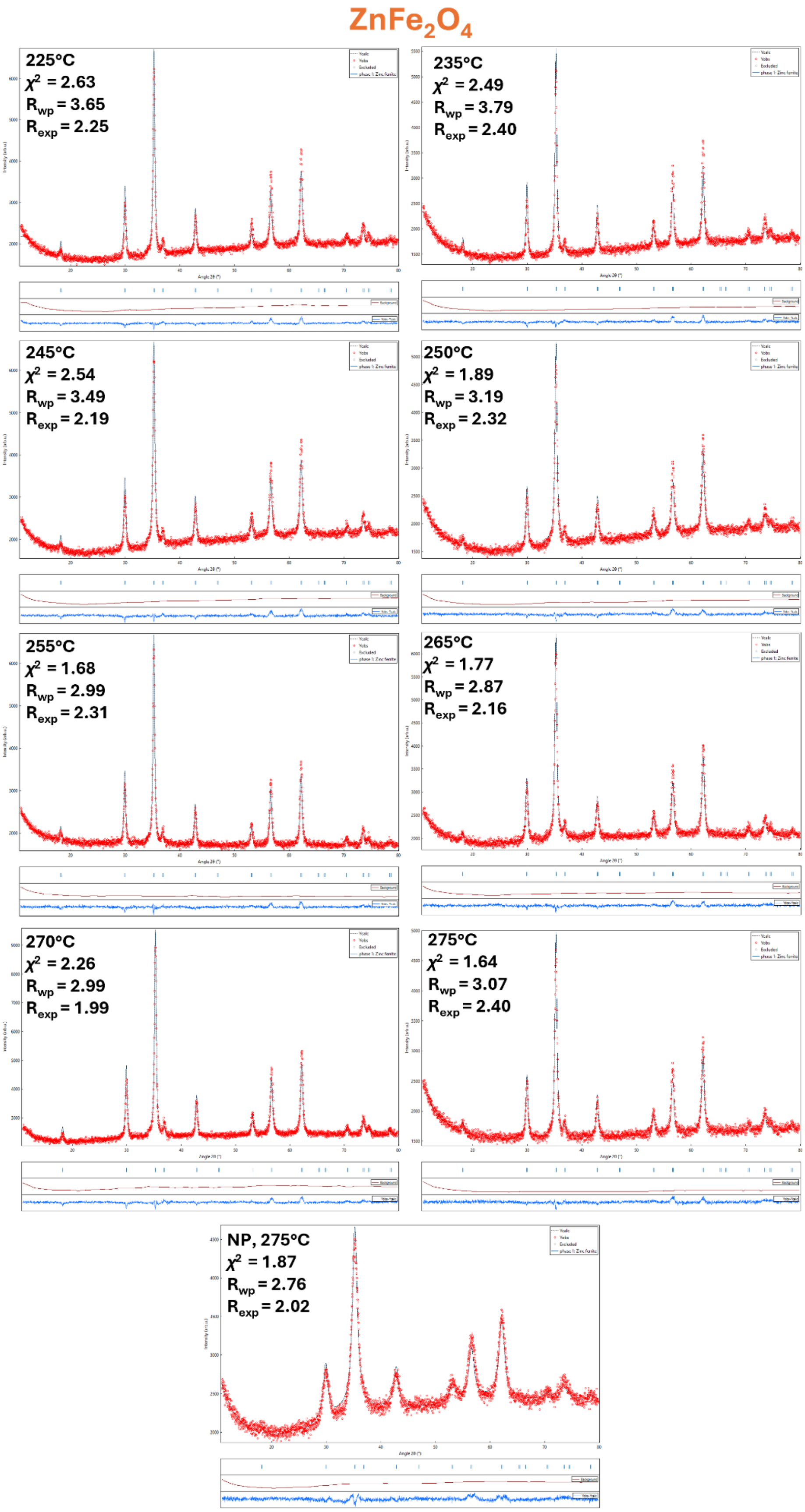

Figure S7. Rietveld refinement results of $ZnFe_2O_4$.

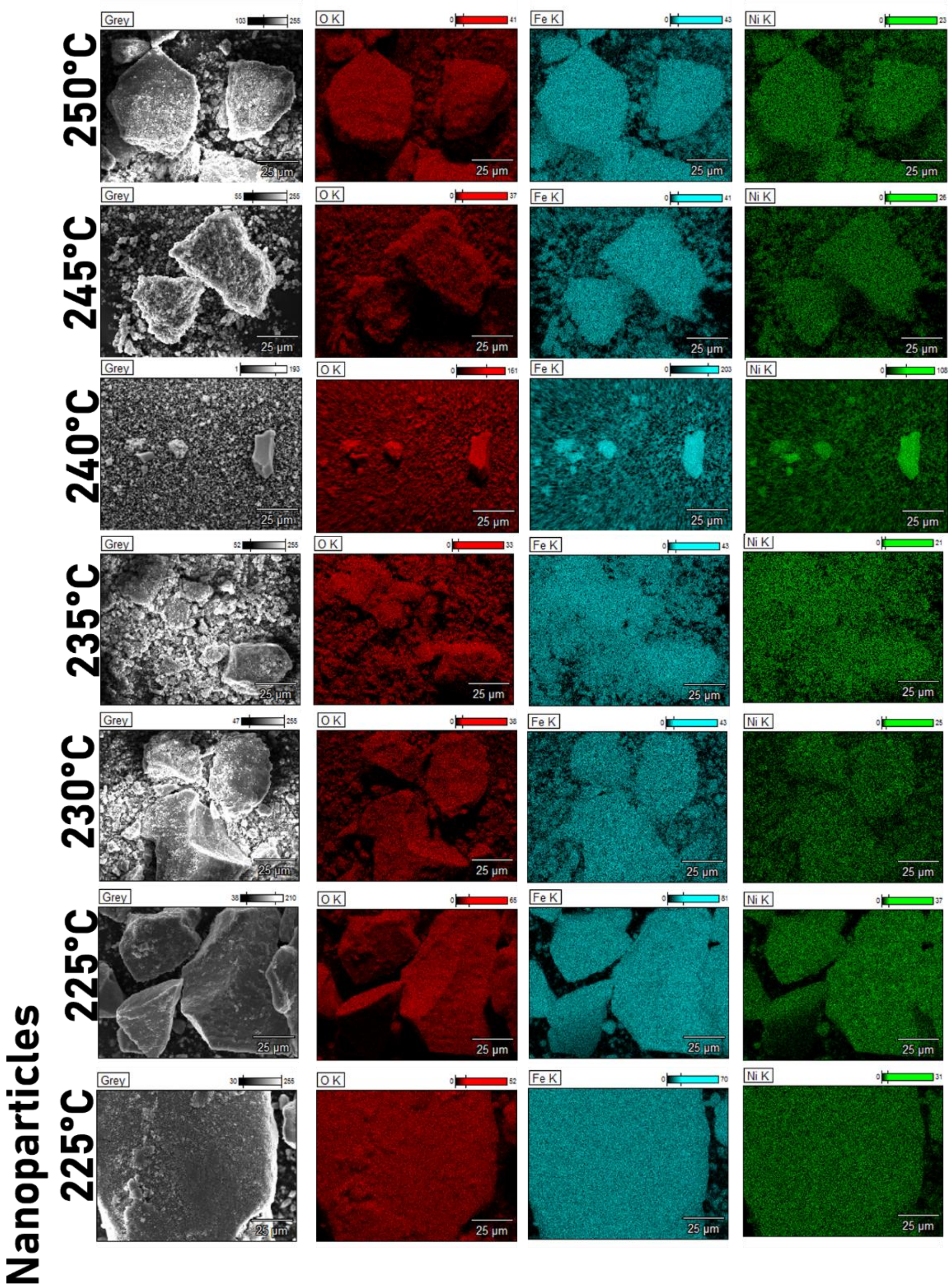

Figure S8. EDXS mapping of $NiFe_2O_4$ prepared at different temperatures.

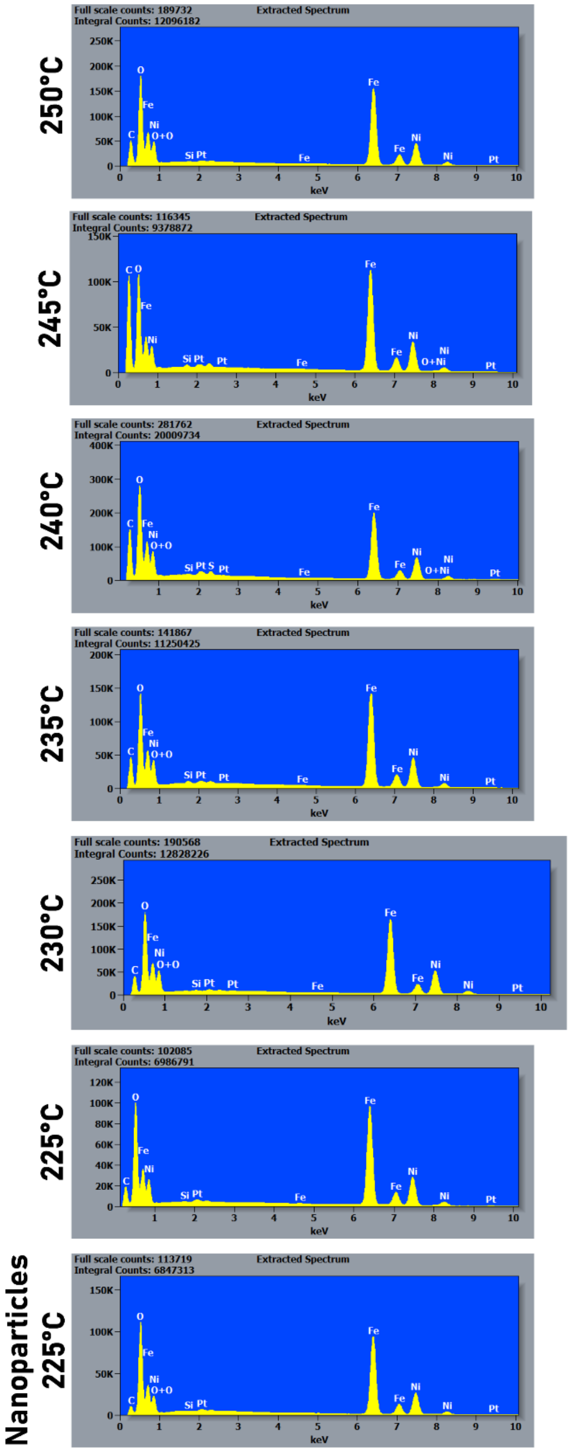

Figure S9. EDX spectra of $NiFe_2O_4$, prepared at different temperatures.

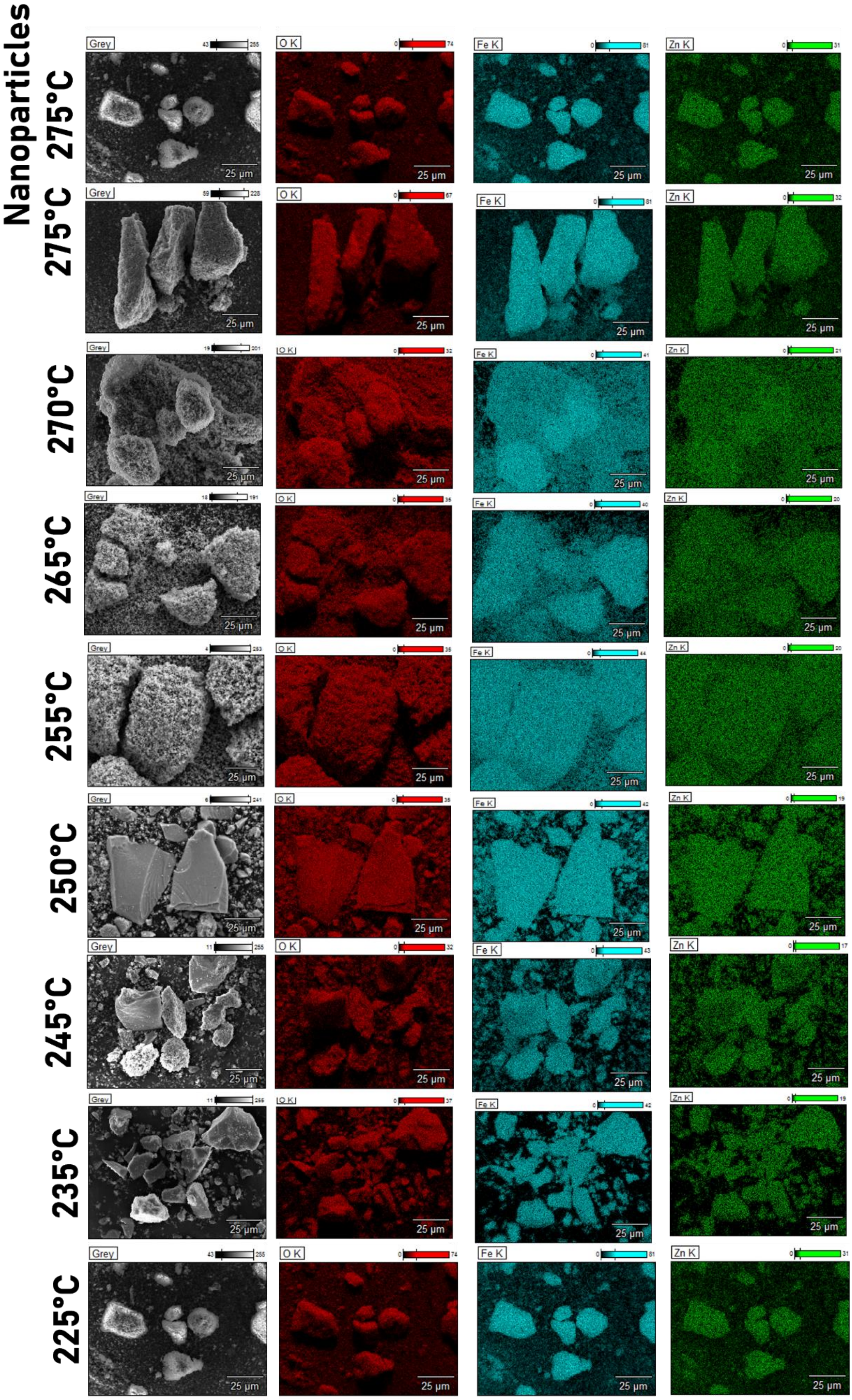

Figure S10. EDXS mapping of $ZnFe_2O_4$ prepared at different temperatures.

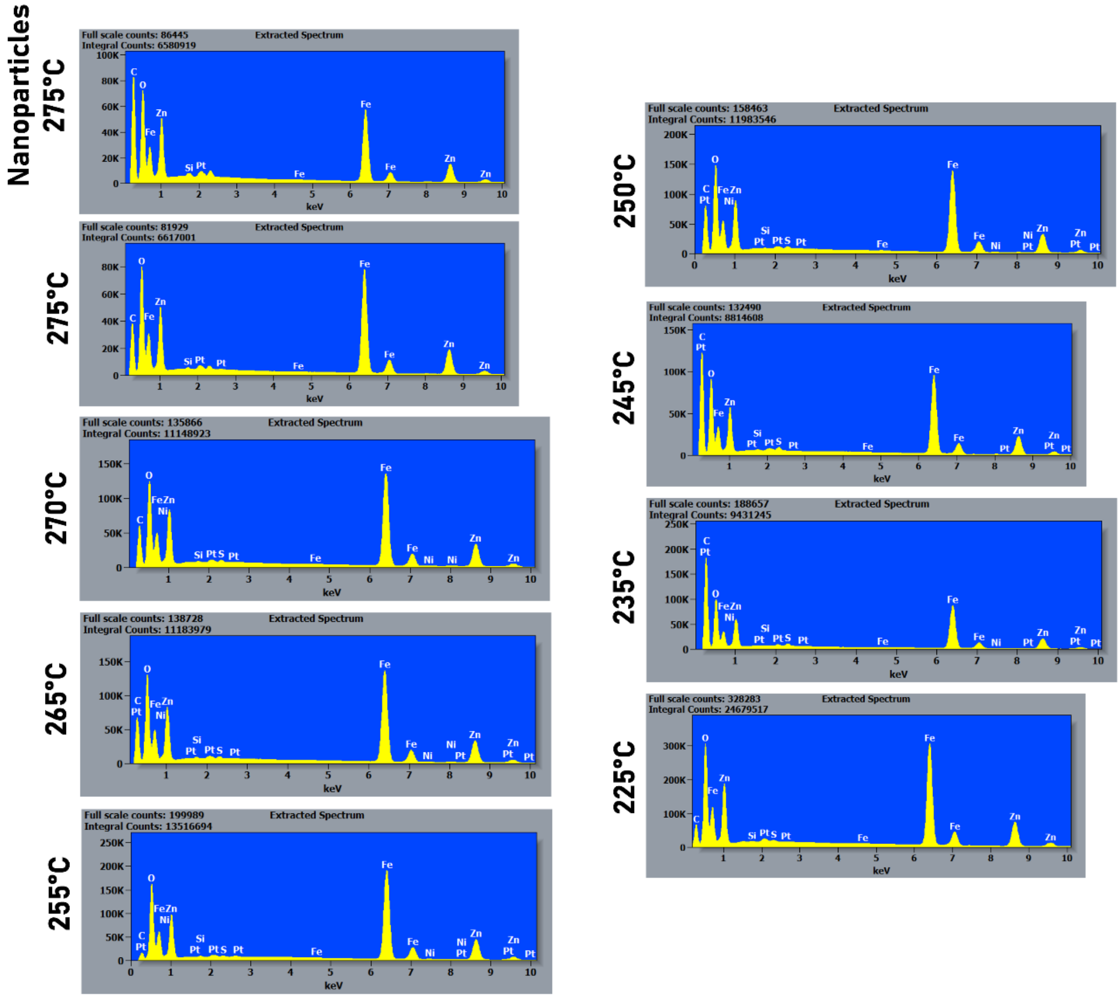

Figure S11. EDX spectra of $ZnFe_2O_4$, prepared at different temperatures.

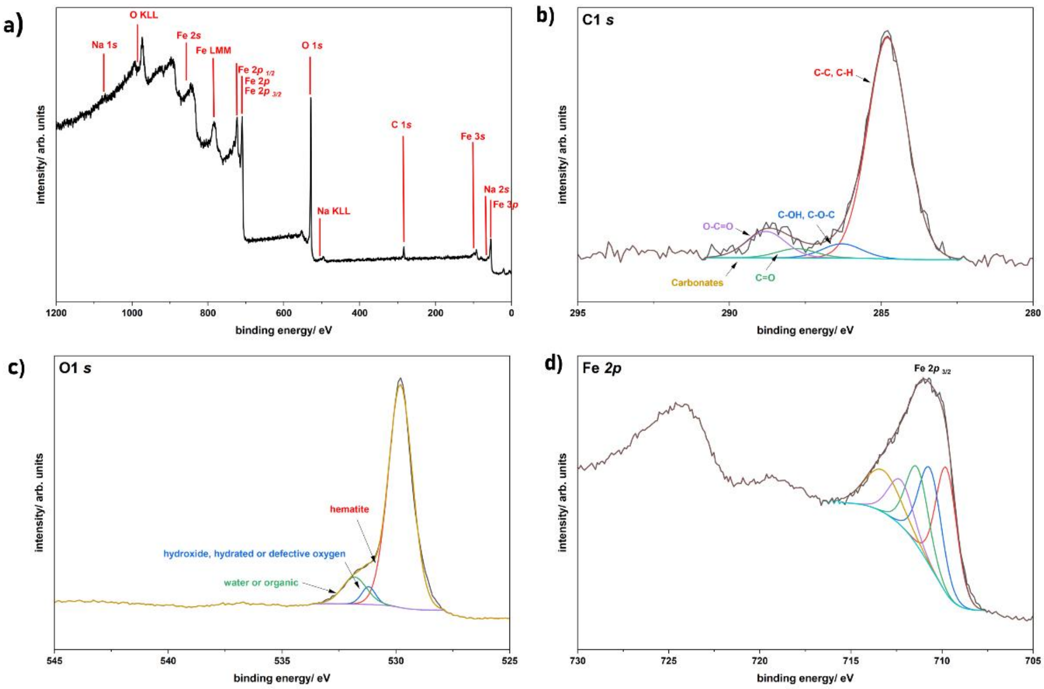

Figure S12. a) XP survey spectrum and b) C 1*s*, c) O 1*s*, d) Fe 2*p* spectrum of α-Fe_2O_3, prepared with P-123®.

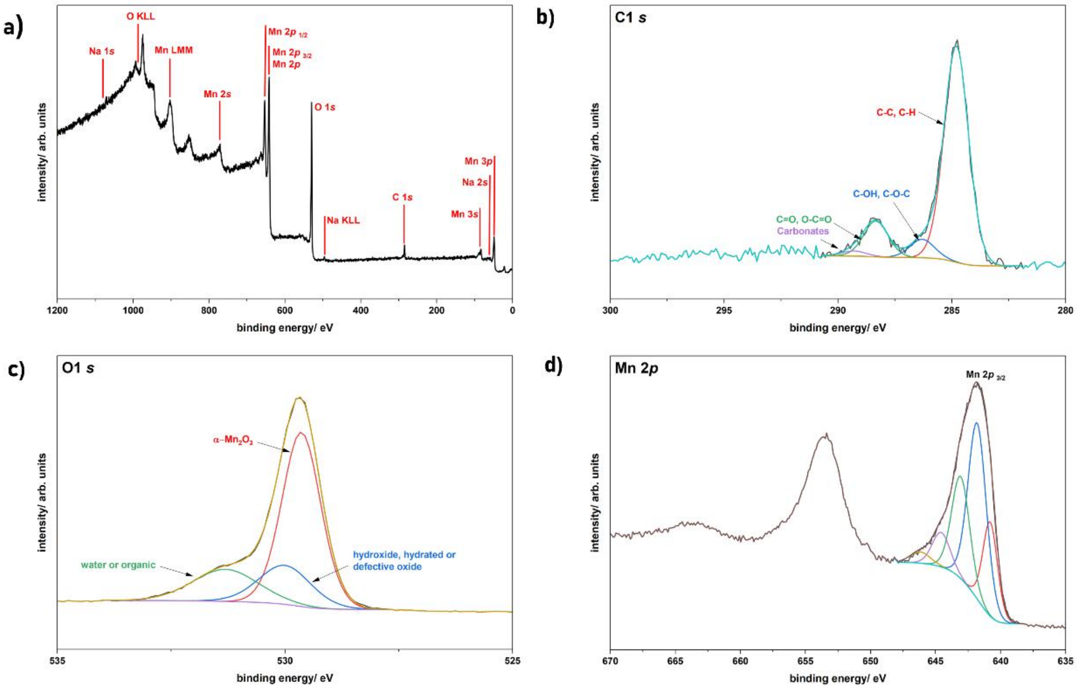

Figure S13. a) XP survey spectrum and b) C 1*s*, c) O 1*s*, d) Mn 2*p* spectrum of α-Mn_2O_3, prepared with P-123®.

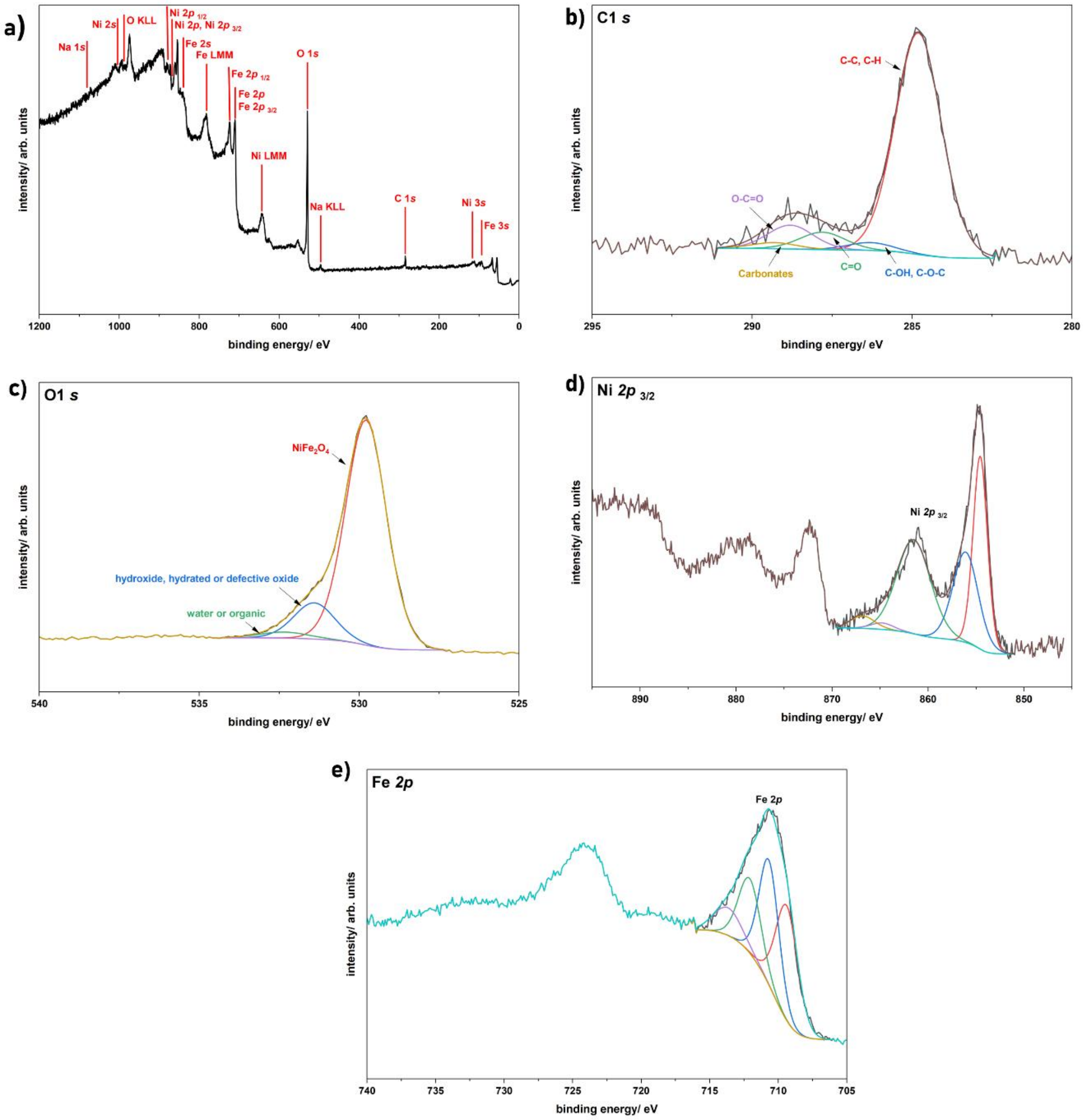

Figure S14. a) XP survey spectrum and b) C 1*s*, c) O 1*s*, d) Ni 2*p* $_{3/2}$, e) Fe 2*p* spectrum of $NiFe_2O_4$, prepared at 240°C.

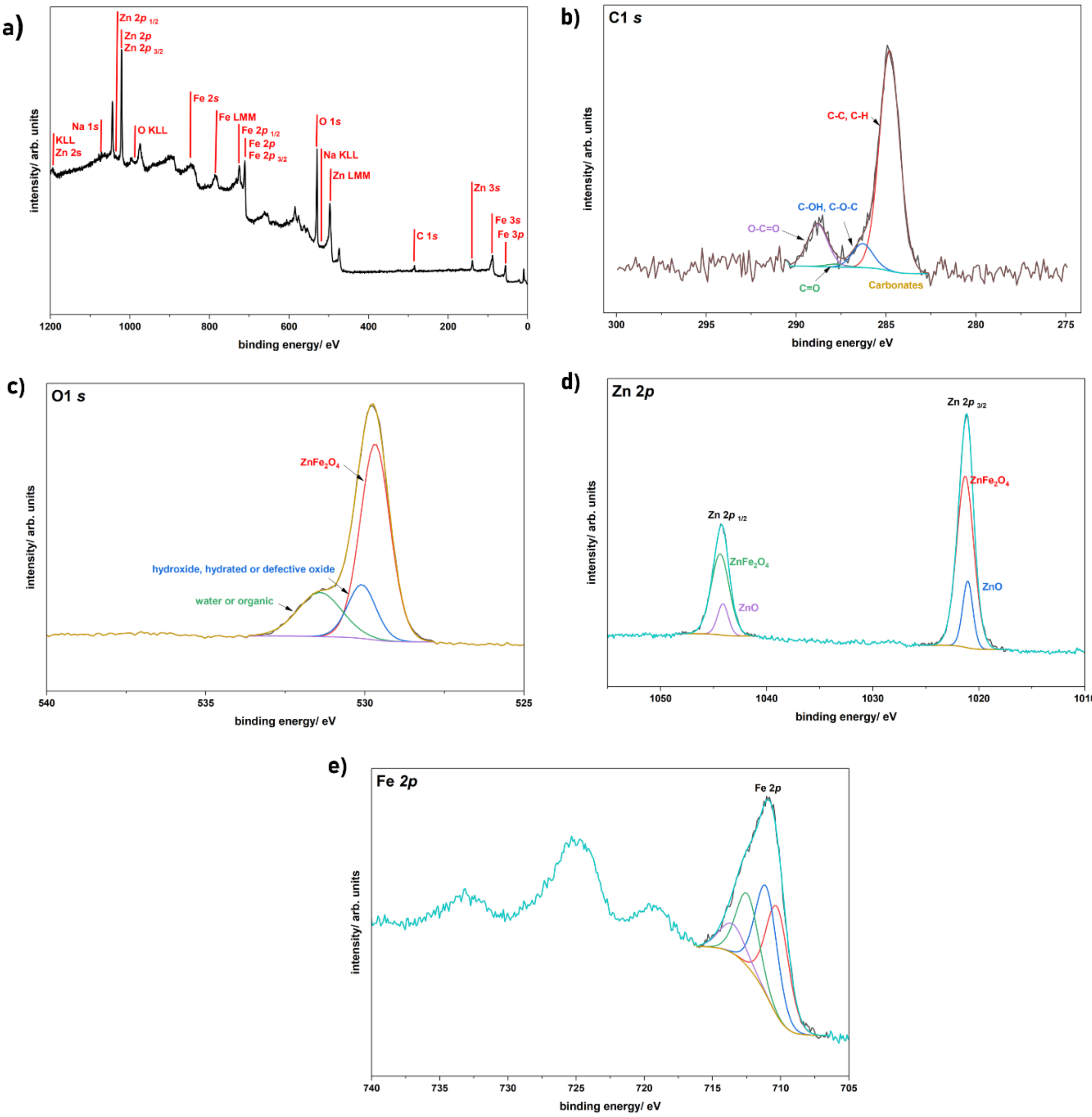

Figure S15. a) XP survey spectrum and b) C 1*s*, c) O 1*s*, d) Zn 2*p*, e) Fe 2*p* spectrum of $ZnFe_2O_4$, prepared at 275°C.

Table S3. Atomic weight percentages of elements, determined by XPS.

	**α-Fe_2O_3**	**α-Mn_2O_3**	**$NiFe_2O_4$**	**$ZnFe_2O_4$**
**C 1*s*/ at%**	13.98	15.41	10.39	9.44
**Fe 2p/ at%**	20.35	-	22.37	17.77
**O 1*s*/ at%**	64.24	54.56	54.73	56.74
**Na 1*s*/ at%**	1.42	1.21	1.41	0.94
**Mn 2*p*/ at%**	-	28.82	-	-
**Ni 2p $_{3/2}$/ at%**	-	-	11.10	-
**Zn 2*p* $_{3/2}$/ at%**	-	-	-	15.11

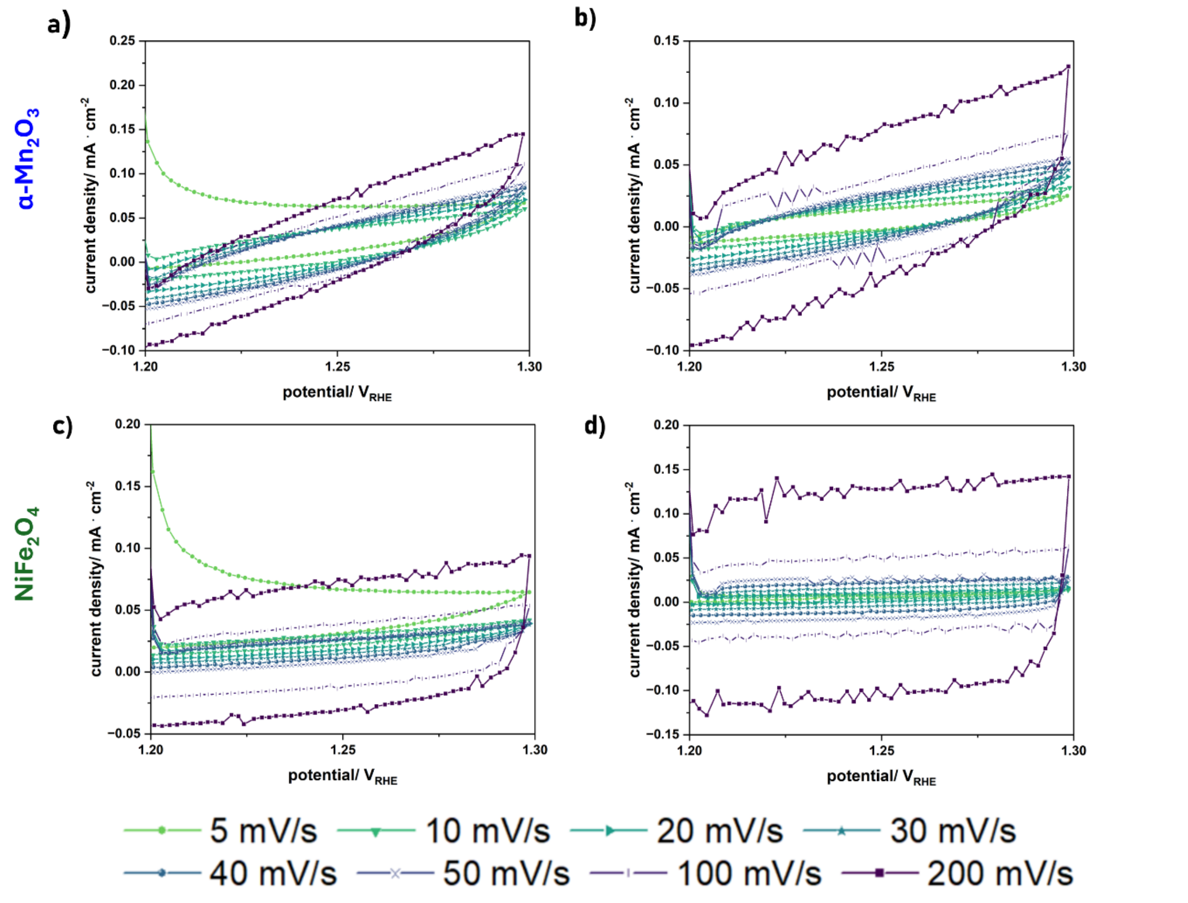

Figure S16. CV-Scans for ECSA calculation with varying scan rate before (left) and after LSV (right) for a), b) α-Mn_2O_3 and c), d) $NiFe_2O_4$.

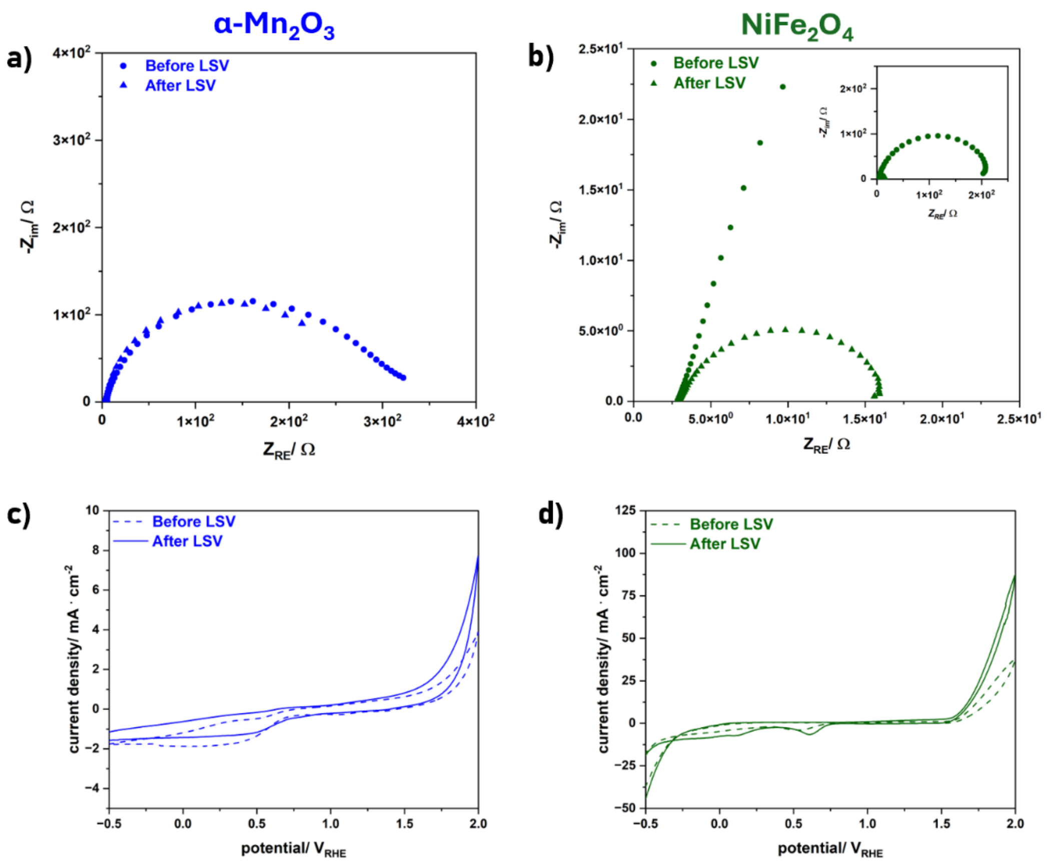

Figure S17. a), b) Nyquist plots before and after LSV at 1.7 V, c), d) CV scans before and after LSV with a scan rate of 20 mV/s.

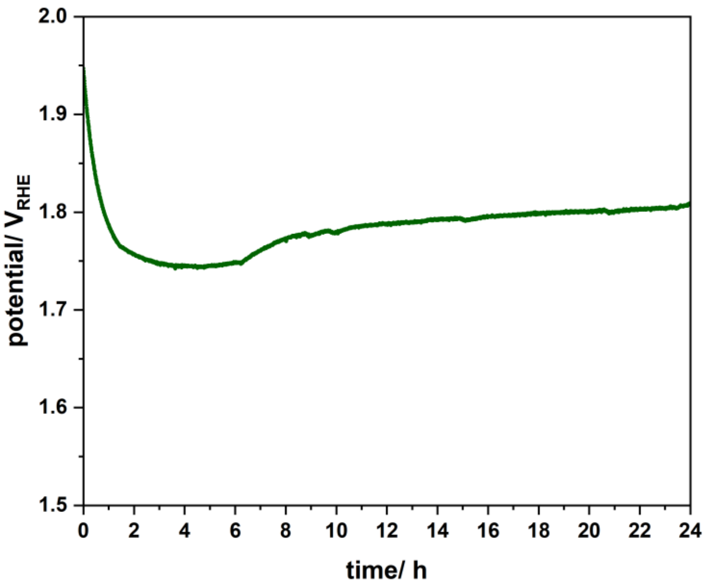

Figure S18. Chronopotentiometric long-term stability tests at applied current (10 mA) of NiFe$_2$O$_4$@carbon paper in 1M KOH.

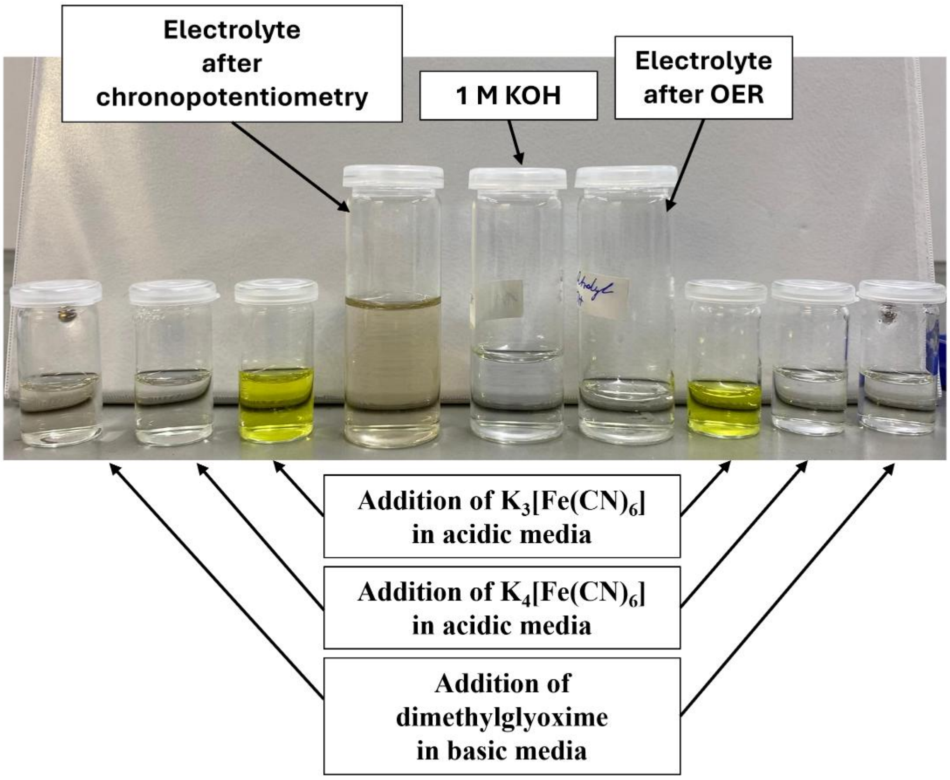

Figure S19. Electrolyte after OER and chronopotentiometry of mesoporous $NiFe_2O_4$ and addition of $K_4[Fe(CN)_6]$, $K_3[Fe(CN)_6]$ and dimethylglyoxime for the detection of Fe^{2+}, Fe^{3+}, Ni^{2+} respectively.

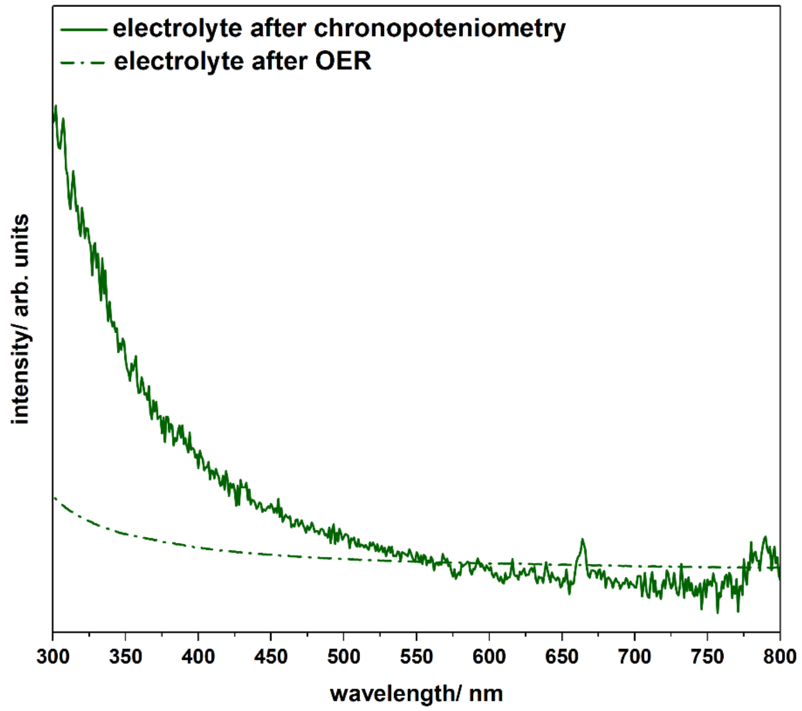

Figure S20. UV-Vis spectra of the electrolyte after OER (dashed line) and after chronopotentiometry (green line) of mesoporous $NiFe_2O_4$.
